# Metabolomic Analysis of *SCD* during Goose Follicular Development: Implications for Lipid Metabolism

**DOI:** 10.3390/genes11091001

**Published:** 2020-08-26

**Authors:** Xin Yuan, Shenqiang Hu, Liang Li, Hehe Liu, Hua He, Jiwen Wang

**Affiliations:** Farm Animal Genetic Resources Exploration and Innovation Key Laboratory of Sichuan Province, College of Animal Science and Technology, Sichuan Agricultural University, Chengdu 611130, China; b20161703@stu.sicau.edu.cn (X.Y.); shenqiang.hu@sicau.edu.cn (S.H.); liliang@sicau.edu.cn (L.L.); liuee1985@sicau.edu.cn (H.L.); hehua@sicau.edu.cn (H.H.)

**Keywords:** Stearoyl-CoA, goose follicular development, lipid metabolism, granulosa cells, *SCD* overexpression, *SCD* knockdown

## Abstract

Stearoyl-CoA desaturase (SCD) is known to be an important rate-limiting enzyme in the production of monounsaturated fatty acids (MUFAs). However, the role of this enzyme in goose follicular development is poorly understood. To investigate the metabolic mechanism of SCD during goose follicular development, we observed its expression patterns in vivo and in vitro using quantitative reverse-transcription (qRT)-PCR. Liquid chromatography-tandem mass spectrometry (LC-MS/MS) was used to determine a cellular model of SCD function in granulosa cells (GCs) via *SCD* overexpression and knockdown. qRT-PCR analysis showed that *SCD* was abundantly expressed in the GC layer, and was upregulated in preovulatory follicles. Peak expression was found in F1 and prehierarchal follicles with diameters of 4–6 mm and 8–10 mm, respectively. We further found that mRNA expression and corresponding enzyme activity occur in a time-dependent oscillation pattern in vitro, beginning on the first day of GC culture. By LC-MS/MS, we identified numerous changes in metabolite activation and developed an overview of multiple metabolic pathways, 10 of which were associated with lipid metabolism and enriched in both the overexpressed and knockdown groups. Finally, we confirmed cholesterol and pantothenol or pantothenate as potential metabolite biomarkers to study SCD-related lipid metabolism in goose GCs.

## 1. Introduction

An important rate-limiting enzyme in lipogenesis is stearoyl-CoA desaturase (SCD), which synthesizes monounsaturated fatty acids (MUFAs) from saturated fatty acids (SFAs) by introducing a cis-double bond into fatty acyl-CoAs [1]. The products of SCD are major substrates for the biosynthesis of endogenous polyunsaturated fatty acids (PUFAs) and complex lipids such as phospholipids, triglycerides, wax esters, and cholesterol esters. [2]. The ratio of SFAs to MUFAs can influence a broad spectrum of cellular functions; thus, the content and distribution of SFAs and MUFAs within the cell must be tightly controlled by SCD [3]. Furthermore, SCD is an endoplasmic reticulum membrane protein, where it undergoes rapid turnover in response to nutritional and hormonal signal variation [4] and has had a vital metabolic function during evolution [5]. Recently, research has revealed the influence of SCD on lipid metabolism, membrane fluidity, and energy metabolism [6,7,8]. Therefore, SCD has been identified as an important metabolic control point and is emerging as a promising therapeutic target for the treatment of obesity, diabetes, and other metabolic diseases [3,9]. Moreover, some studies point to SCD as a main factor in the control of cancer cell growth [10,11].

Lipid metabolism has an important biological role in living cells [12], including follicular cells. Investigations of lipid profiles in both follicular cells (including cumulus, GCs, and theca cells (TCs)) and mass spectrometry (MS) of follicular fluid suggest that lipid metabolism is pivotal for oocyte maturation and follicular development [13,14]. Moreover, as the capacity of oocytes to utilize glucose as the main energy source is limited [15], lipid metabolism in GCs of follicles is considered indispensable for oocyte maturation in cattle, sheep, and humans [16,17,18]. In a previous study, we confirmed for the first time that de novo lipogenesis occurs in goose GCs [19]. More importantly, we identified miRNA–mRNA interaction pairs related to the regulation of lipid metabolism during goose follicular development [20]. Although studies have demonstrated that SCD is an important rate-limiting enzyme in lipid metabolism, research into avian follicular development and oocyte maturation, which have some peculiarities, is currently lacking. It has been speculated that endogenous MUFA synthesis yields a source that is distinct from the dietary MUFA pool and acts as a metabolic switch that influences the balance of energy storage versus energy oxidation [21,22]. Nevertheless, the mechanism by which energy status affects avian ovarian follicular selection, as well as follicular recruitment and growth, has not been thoroughly investigated and is not yet fully elucidated.

**Figure 1:**
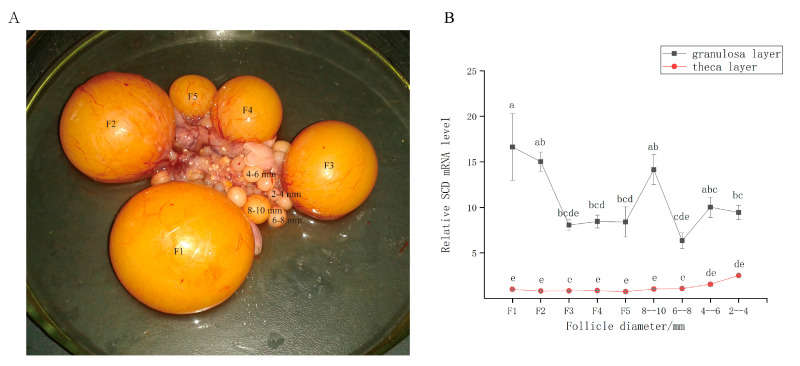
(**A**) The goose ovary contains a hierarchy of large preovulatory follicles and a small cohort of prehierarchal follicles. (**B**) Relative expression level of Stearoyl-CoA desaturase (SCD) in granulosa layer and theca layer during follicle development in vivo. The data are represented as the mean ± SD (*n* = 3); the data were analyzed by ANOVA and Tukey’s test. The lowercase letters indicate significant differences between different granulosa cell (GC) layers and theca cell (TC) layers (*p* < 0.05).

The indigenous Tianfu goose (*Anas cygnoides*) is a commercially important farm animal in southern China. However, its poor egg-laying performance is a hindrance to the industry [23]. The goose ovary contains a hierarchy of large preovulatory follicles (designated F1–F5) and a small cohort of prehierarchal follicles (Figure 1A). The largest follicle (F1) ovulates before the others, and F2 then replaces F1 as the dominant follicle and ovulates 48 h later. This process is successively repeated by F3–F5. Unlike in mammals, however, all follicles within this cohort do not undergo atresia [24]. The prehierarchal follicles are categorized by size (2–4, 4–6, 6–8, and 8–10 mm in diameter). Follicular recruitment from the prehierarchal cohort is initiated in the 4–6 mm follicle, and follicular selection within the preovulatory hierarchy occurs in a cohort of prehierarchal follicles (8–10 mm follicle). Understanding the mechanisms through which the processes associated with follicular recruitment and selection occur has great value for follicular development research [25].

Metabolomics is defined as the characterization and quantification of small molecules that are mediators and products of metabolism, with the aim of monitoring metabolism and its fluctuations in biological samples [26]. Therefore, metabolomic studies provide insights into variations in the levels of endogenous metabolites as a living system responds to biological stimuli or genetic modification [27]. The overall goal of this study was to elucidate the underlying metabolites and pathways that are activated in goose GCs during follicular development, using a metabolomic approach by creating a cellular model of SCD function. We analyzed the SCD expression patterns in goose follicles in vivo and in vitro by quantitative reverse-transcription PCR (qRT-PCR), and applied liquid chromatography-tandem MS (LC-MS/MS) to investigate the effects of metabolic alterations, particularly those related to lipids, on goose follicular development.

## 2. Materials and Methods

### 2.1. Ethics Statement

All experimental procedures that involved animal manipulation were approved the Committee of the School of Farm Animal Genetic Resources Exploration and Innovation Key Laboratory, College of Animal Science and Technology, Sichuan Agricultural University, under permit no. DKY20170913, and were performed in accordance with the Regulations for the Administration of Affairs Concerning Experimental Animals (China 1988). All efforts were made to minimize animal suffering, conducted in accordance with the requirements of the Beijing Animal Welfare Committee.

### 2.2. Goose Follicle Collection and Primary GC Culture

Geese (from a maternal line of the Tianfu goose) were raised under natural temperature and light conditions at the waterfowl breeding experimental farm of Sichuan Agricultural University. For follicle collection, six geese showing regular laying schedules were randomly selected, all sacrificed 2 h after oviposition via post-anesthesia exsanguination. Ovarian follicles were collected from the goose abdominal cavities and divided into prehierarchal follicles according to their sizes (<2, 2–4, 4–6, 6–8, and 8–10 mm in diameter) and a hierarchy of preovulatory follicles (F5, F4, F3, F2, and F1) according to previously reported nomenclature [28]. The granulosa layer and theca layers were isolated as previously described [29]. Then they were stored at −80 °C for RNA extraction.

For primary GC culture, the granulosa layer was dispersed by incubation in 0.1% type II collagenase (Sigma, St. Louis, MO, USA) for 10 min in a 37 °C water bath. After incubation, the cells were dispersed and pelleted by centrifugation at 1000× *g* for 10 min. Then, the cells were re-suspended in 3 mL of fresh basic medium without collagenase and centrifuged. The washing procedure was repeated twice. The GCs were dispersed in Dulbecco’s modified Eagle medium (DMEM) supplemented with 1% antibiotic/antimycotic solution (Solarbio, Beijing, China) and 3% fetal bovine serum (Gibco, Waltham, MA, USA). The viability of all GCs was greater than 90%. Cells were incubated in a water-saturated atmosphere of 95% air and 5% CO_2_ at 37 °C in an incubator (Thermo, Waltham, MA, USA).

### 2.3. RNA Extraction and qRT-PCR

Total RNA was extracted from each sample using Trizol reagent (Invitrogen, Waltham, MA, USA) according to the manufacturer’s instructions. The first-strand cDNA was synthesized from total RNA (1 μg) using a cDNA synthesis kit (Takara, Shiga, Japan). qRT-PCR was conducted using synthesized cDNA with the SYBR PrimeScript RT-PCR kit (Takara, Shiga, Japan) in the CFX96™ Real-Time System (Bio-Rad, Hercules, CA, USA); primers used for qRT-PCR are listed in Table S1. Relative mRNA level was determined using the 2^(−ΔΔCt)^ method [30]. *β-actin* and *GAPDH* mRNA levels were used to normalize mRNA levels.

### 2.4. Modulation of SCD with Small Interfering RNA

Specific small interfering RNA (siRNA), used to silence *SCD* expression, was synthesized by GenePharma (Shanghai, China) and was transfected into GCs using the Lipofectamine™ RNAiMAX Transfection Reagent (Invitrogen Co., Waltham, MA, USA) according to the manufacturer’s recommendations. Briefly, the GCs were grown in medium (DMEM) without antibiotics 1 day before the experiment. On the day of transfection, pre-prepared siRNA–RNAiMAX complexes were incubated for 5 min at room temperature. The cells that we had prepared for *SCD* knockdown were removed from their medium and placed onto medium that was free of serum or antibiotics. The cells were then incubated with the siRNA–RNAiMAX complexes at 37 °C for 24, 48, and 72 h. siRNA was delivered to cells at a final concentration of 20 nmol/L. Cells were collected for mRNA analysis to verify gene knockdown. Scrambled siRNA was used as a nonspecific negative control. The sequences for the siRNAs used are listed in Table 1.

### 2.5. Overexpression of SCD with Recombinant Vector

To generate GFP-SCD, the RNA was obtained from normal goose ovarian tissue and used to generate cDNA clones of the *SCD* gene. A 981-bp cDNA fragment (GenBank Accession No. XM_013201691.1) was amplified using primers (Table 1) capped with *Xho*I and *Hin*dIII recognition sequences. This fragment was then inserted to construct the pEGFP-N1 plasmid. The construct was confirmed by enzymatic digestion and DNA sequencing. In the transient transfection experiment, 1µg of the plasmid DNA was transfected into 1 × 10^6^ GCs in six-well dishes using Lipofectamine^®^ 3000 (Invitrogen Co) according to the manufacturer’s instructions; a GFP vector and an empty control served as negative controls. The expression levels of *SCD* mRNA were detected 24, 48, and 72 h later to evaluate transfection efficiency.

### 2.6. Determination of SCD Activity

The SCD activity was measured using the Goose Stearoyl-CoA Desaturase Activity Assay Kit (NJJCBIO, Nanjing, China). Briefly, each cell culture medium was diluted five times with sample diluent; 50 µL of the resultant dilution was then added to the enzyme label plate. Plates were incubated at 37 °C for 30 min, washed five times with wash buffer, and air-dried at room temperature. Standard reagent (50 µL) was added to the plates, which were then washed five times. After adding 50 µL reagent A and 50 µL reagent B, the plates were incubated at 37 °C in the dark for 10 min. Finally, 50 µL stop buffer was added; the optical density (OD) value was measured using the automatic enzyme immunoassay analyzer at a wavelength of 450 nm and calculated.

### 2.7. Cell Sample Preparation for Extraction

In total, 18 samples, consisting of three biological replicates, were randomly and independently analyzed to reduce analysis bias. After the samples were thawed on ice, 1 mL pre-cooled extractant (70% methanol aqueous solution) was added and vortexed for 1 min. The mixture was placed in liquid nitrogen for 3 min, removed from ice for 3 min, and vortexed for an additional 2 min. This procedure was repeated three times. The mixture was again centrifuged at 12,000 rpm at 4 °C for 10 min. Finally, the supernatant was injected into the sample bottle until use.

### 2.8. LC-Electrospray Ionization (ESI)-QTRAP-MS/MS) Analysis

Samples were analyzed using the LC-ESI-QTRAP-MS/MS system. (UPLC, Shim-pack UFLC SHIMADZU CBM A system; MS, QTRAP^®^ System). A 2-μL aliquot of the resulting supernatant was injected into a UPLC column (2.1 × 100 mm^2^, 1.8 μm) using a linear gradient (95:5 *v*/*v* at 0 min, 5:95 *v*/*v* at 11.0 min, 5:95 *v*/*v* at 12.0 min, 95:5 *v*/*v* at 12.1 min, and 95:5 *v*/*v* at 14.0 min) at a flow rate of 0.4 mL/min, held at 40 °C. The binary gradient elution system consisted of water (0.04% acetic acid) and acetonitrile (0.04% acetic acid). The mass spectrometric data were collected using a triple quadrupole linear ion trap mass spectrometer system equipped with an ESI Turbo Ion-Spray interface, operating in positive and negative ion mode, and controlled by Analyst 1.6.3 software (Sciex).

The mixed quality control (QC) samples comprised a mixture of extracts from each sample, which were analyzed using the same method as that used for the experimental samples. The mixed QCs were injected after every five experimental samples throughout the analytical run, providing a set of data from which repeatability could be assessed.

### 2.9. Qualitative and Quantitative Analysis of Metabolites

The raw LC-ESI-QTRAP-MS/MS data files were processed using R platform (Scripps, La Jolla, CA, USA) to perform peak picking, alignment, integration, and retention time (RT) alignment. Integration and correction of chromatographic peaks was performed using Progenesis QI software (Waters Co., Milford, MA, USA). The corresponding relative metabolite contents were represented as chromatographic peak area integrals. In addition, potential metabolites were identified using public databases, including the Human Metabolome Database (http://www.hmdb.ca), Metlin (https://metlin.scripps.edu), and MassBank (http://www.massbank.jp).

### 2.10. Data Processing and Analysis 

Metabolites were used for hierarchical clustering analysis and heat map analysis, which were conducted using R package, version 3.3.1. Subsequently, principal component analysis (PCA) and orthogonal correction partial least squares discriminant analysis (OPLS-DA) were conducted using SIMCA-P14.0 software (Umetrics, Umeå, Sweden) to process data. Significant differences between metabolites of experimental and control groups were identified using variable importance in projection (VIP) from OPLS-DA (VIP > 1). Student’s *t*-test was applied to calculate the statistical significance (P-value) of differential metabolites obtained from OPLS-DA modeling at the univariate analysis level. The Kyoto Encyclopedia of Genes and Genomes (KEGG) database (http://www.genome.jp/kegg/) was used to identify enriched pathways of the differing metabolites.

The qRT-PCR and enzyme activity data from the three independent biological replicates were analyzed by one-way ANOVA using SPSS 19.0 (SPSS Inc., Chicago, IL, USA) for the comparison of multiple means. All data are expressed as mean ± SD, and significance was assumed at *p* < 0.05. Further, all data were illustrated using GraphPad Prism 6.01 (GraphPad Software, San Diego, CA, USA).

## 3. Results

### 3.1. SCD is Expressed during Follicular Development In Vivo

We performed qRT-PCR analysis to detect the expression patterns of *SCD* in the granulosa and theca layers during follicular development (Figure 1B). The results showed that *SCD* was primarily expressed in the granulosa layer and weakly expressed in the theca layer. The *SCD* gene was upregulated in the granulosa layers of the large preovulatory follicles; expression was highest in F1. In the prehierarchal follicles, *SCD* expression was greatest in the 4–6 mm and 8–10 mm size categories.

### 3.2. SCD is Expressed during GC In Vitro Culture

**Figure 2:**
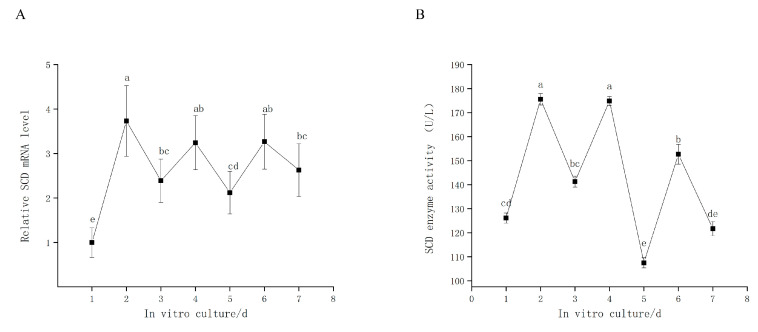
(**A**) Relative expression level of SCD in goose granulosa cells (GCs) cultured in vitro for 7 days. (**B**) SCD enzyme activity. The data are represented as the mean ± SD (*n* = 3); the data were analyzed by ANOVA and Tukey’s test. The lowercase letters indicate significant differences in cultured goose GCs for 7 days in vitro (*p* < 0.05).

The goose GCs were cultured in vitro for 7 d; culture medium was replaced with fresh medium every 2 d. We found that *SCD* is expressed in a time-dependent oscillation pattern that begins on the first day of culturing (Figure 2A). Our subsequent investigation of SCD activity, as measured by the assay kit (Figure 2B), revealed a trend similar to that of the relative *SCD* level.

### 3.3. A GC Cellular Model of SCD Function

**Figure 3:**
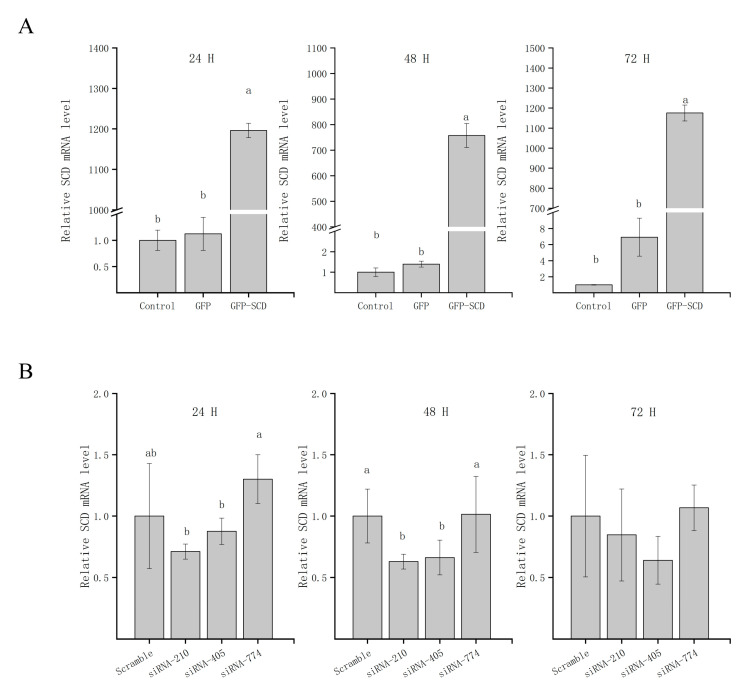
The detection of SCD transfection efficiency. (**A**) Overexpressed-transfected cells were collected at the indicated time points after transfection for qRT-PCR analyses. (**B**) siRNA-transfected cells were collected at the indicated time points after transfection for qRT-PCR analyses. The data are represented as the mean ± SD (*n* = 3); the data were analyzed by ANOVA and Tukey’s test. The lowercase letters indicate significant differences between experimental groups and control groups (*p* < 0.05).

To directly analyze the functional impact of *SCD* in GCs, we generated GCs that transiently overexpress *SCD* (referred to as GFP-SCD) and confirmed *SCD* expression by qRT-PCR (Figure 3A). After 24, 48, and 72 h of transfection, higher expression levels of *SCD* were detected in the transfected group (*p* < 0.05) than in the GFP vector group or the empty control group. We further conducted knockdown studies using siRNA transfection, as shown in Figure 3B. The expression of *SCD* was reduced after specific SCD-siRNA transfection compared to that after transfection with scrambled siRNA. The exceptions were siRNA-774, siRNA-210, and siRNA-405, all of which resulted in reduced *SCD* expression (*p* < 0.05) after 24, 48, and 72 h of transfection. An *SCD*-overexpression group (referred to as S), the GFP vector group (referred to as G), the control group (referred to as N), two independent siRNA groups (siRNA-210 and siRNA-470, referred to as T and F, respectively), and a scrambled siRNA group (referred to as C) were selected for further LC-MS/MS analysis.

### 3.4. Metabolite Differences in N vs. S, G vs. S, C vs. T, and C vs. F Comparisons

**Figure 4:**
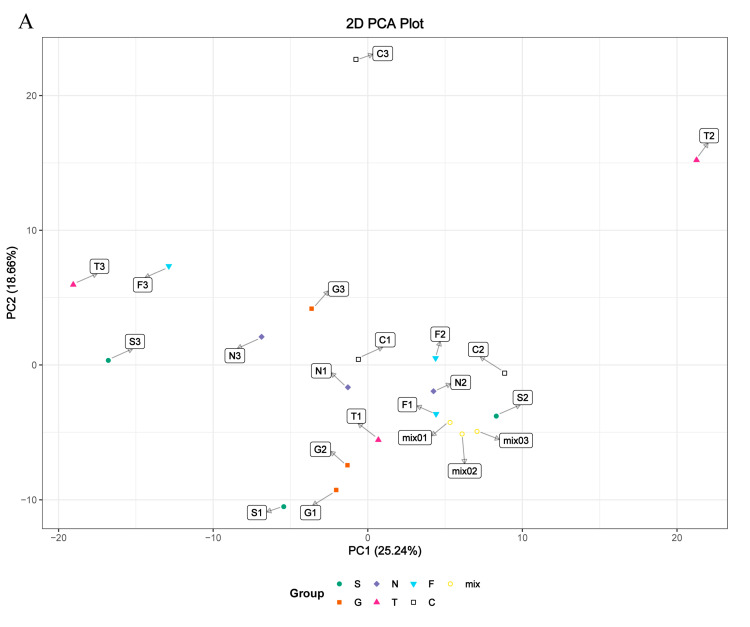

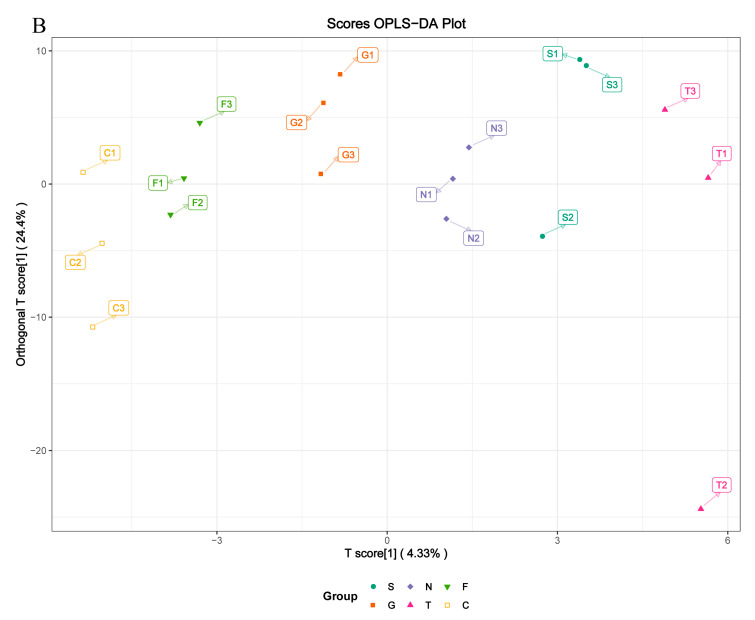
(**A**) Metabolic differences in GC cellular model is highlighted in the principal component analysis (PCA) score plot. The overexpressed SCD group is denoted S, the GFP group is denoted G, and the control group is denoted N; the siRNA-210 group is denoted T, the siRNA-470 group is denoted F, and the scrambled siRNA group is denoted C. For the PCA, the first component accounts for 25.24% of overall variability and the second component accounts for 18.66% of overall variability. (**B**) Orthogonal correction partial least squares discriminant analysis (OPLS-DA) of the metabolites from each group. PCA and OPLS-DA models demonstrating a separation between each group.

**Figure 5:**
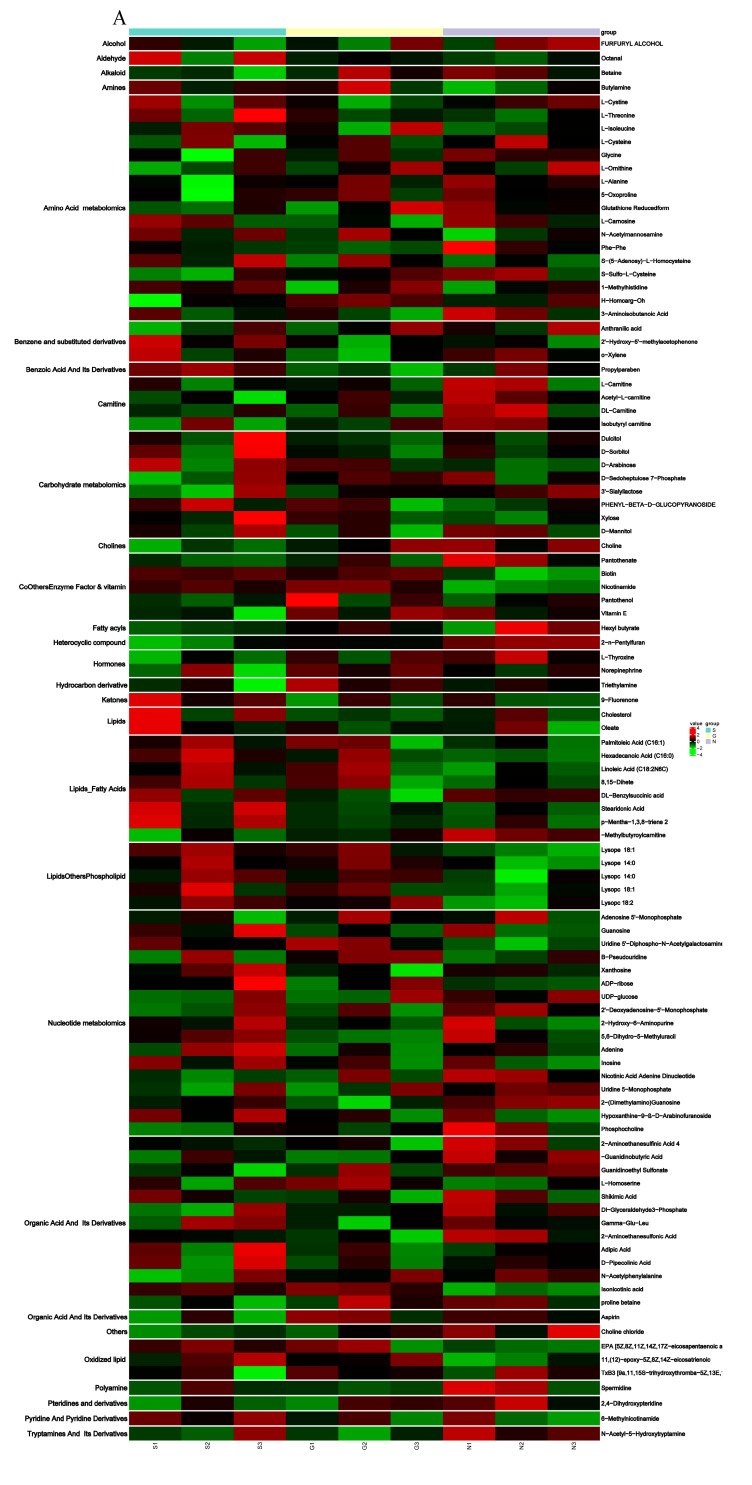

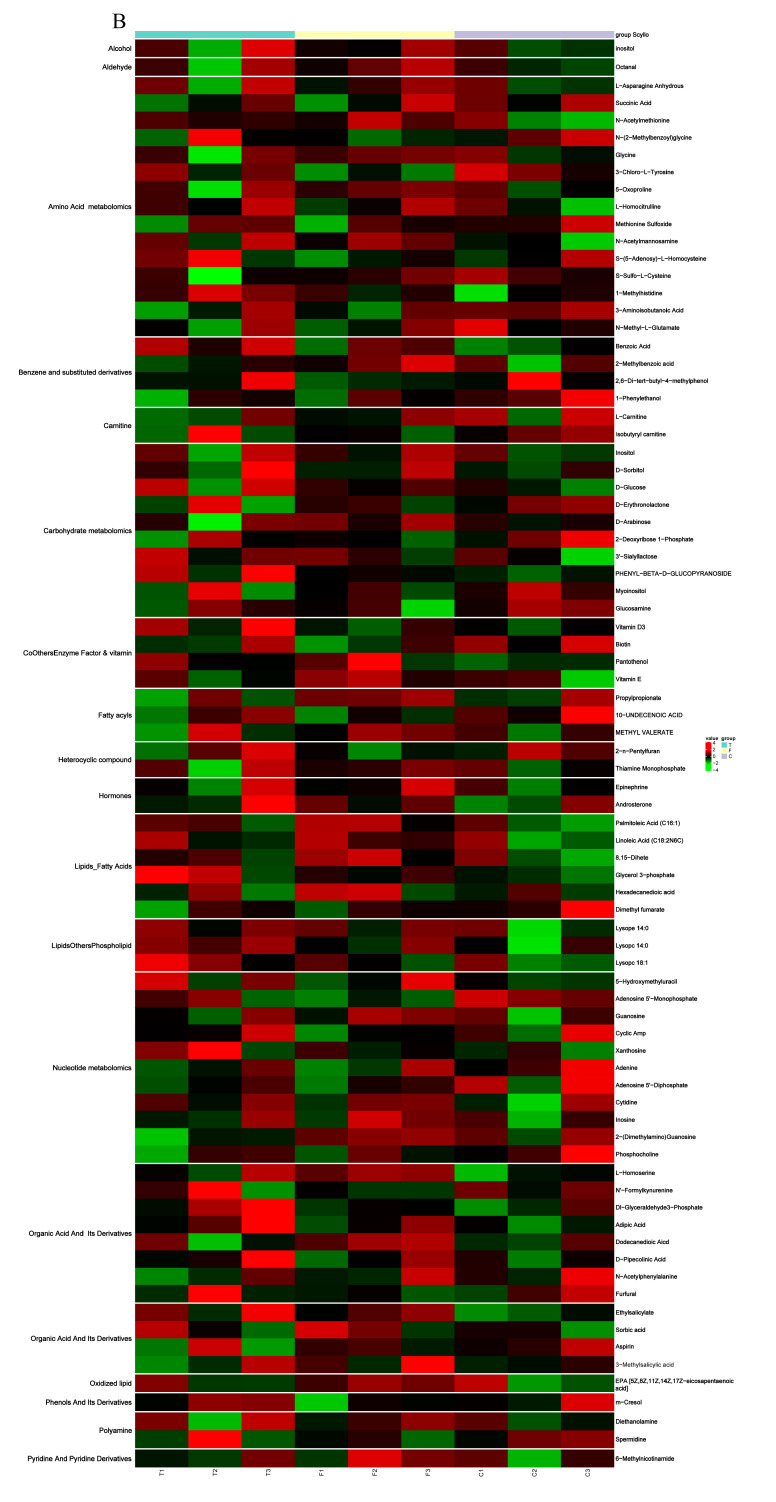
(**A**) Heat map from the hierarchical clustering of differential metabolites in overexpressed SCD group and control group. (**B**) Heat map from the hierarchical clustering of differential metabolites in knockdown SCD group and control group. The scaled expression by row (metabolites) is shown as a heat map and is reordered by a hierarchical clustering analysis (Pearson’s distance and Ward’s method) on both rows and columns. Significant differential metabolites between overexpressed group and knockdown group were identified with cutoff values of a VIP > 1, FC > 1.2. The color scale indicates the relative amounts of metabolites: red, higher levels; green, lower levels; black, unchanged.

**Figure 6:**
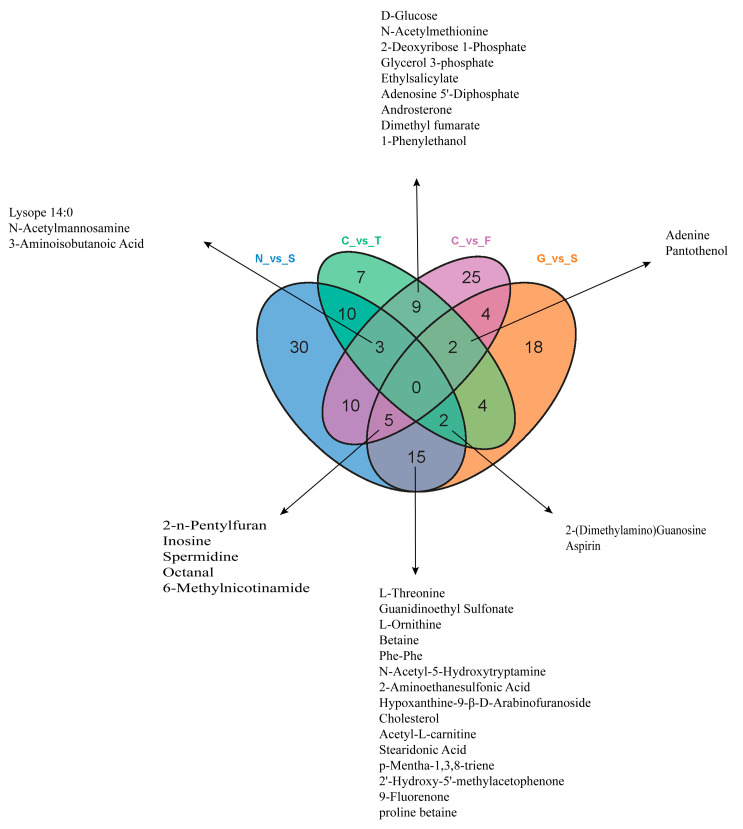
Venn diagram of overlapping and unique metabolites altered in each group. A total of 22 metabolites were overlapping in the N vs. S and G vs. S comparisons. A total of 14 metabolites overlapped in the C vs. T and C vs. F comparisons (Table S2).

Based on the cell transfection efficiency in both the *SCD*-overexpressing and knockdown groups, 10 million cells from each group were collected 48 h after transfection. Metabolite detection was performed using three parallel groups. A total of 333 intracellular metabolites were determined; these included amino acids, lipids, fatty acids, nucleotides, and organic acids. LC-MS/MS analysis was conducted to ensure the accuracy of the results. A complete heatmap of abundant metabolites is presented in Figure S1. As shown in Figure 4, the PCA and OPLS-DA score plots that we constructed using the acquired metabolomic data set revealed that the structure and quality of the data represented the close relationships among the biological replicates and that the samples were distinguished by better clustering and separation between the experimental and control groups. We employed unsupervised hierarchical clustering of significantly different metabolite sets to determine differences between the overexpressing and knockdown groups (Figure 5). A total of 75 metabolites showed significant differences in the N vs. S comparison (41 upregulated and 34 downregulated) based on fold-change analysis (VIP > 1, fold-change (FC) > 1.2), 50 metabolites showed significant differences in the G vs. S comparison (17 upregulated and 33 downregulated), 37 showed significant differences in the C vs. T comparison (14 upregulated and 23 downregulated), and 58 showed significant differences in the C vs. F comparison (24 upregulated and 34 downregulated; Table S2). We arranged the 20 metabolites showing the greatest significant differences according to the log2^FC^ of each group and found that cholesterol showed the greatest change in the overexpressing group, whereas pantothenol showed the greatest change in the knockdown group (Figure S2). To examine the effects of *SCD* overexpression and knockdown on the GC metabolome, we compared differences in metabolites in each group (Figure 6). We found an overlap of 22 metabolites (seven upregulated and ten downregulated; however, Phe-Phe, N-acetyl-5-hydroxytryptamine, 2-aminoethanesulfonic acid, 2-(dimethylamino)guanosine, and spermidine showed the opposite trend) between N vs. S and G vs. S comparisons, as well as an overlap of 14 metabolites (six upregulated and eight downregulated) between C vs. T and C vs. F comparisons.

### 3.5. Pathway Analysis of Differentially Abundant Metabolites

**Figure 7:**
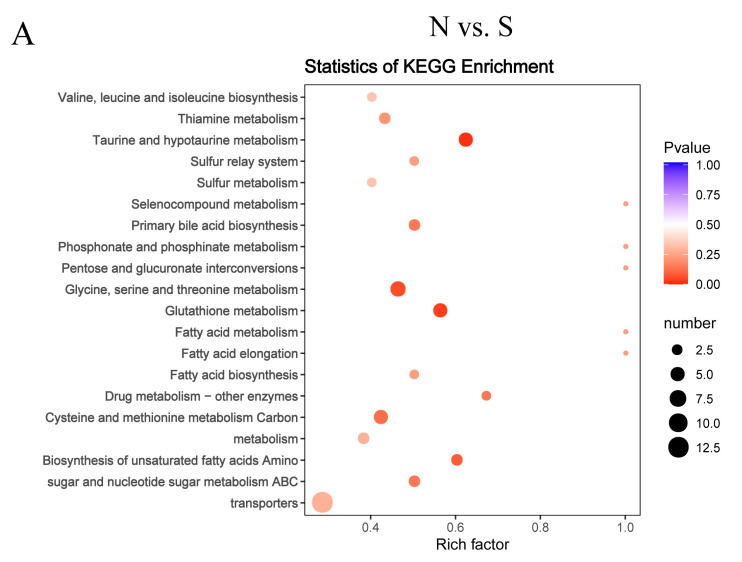

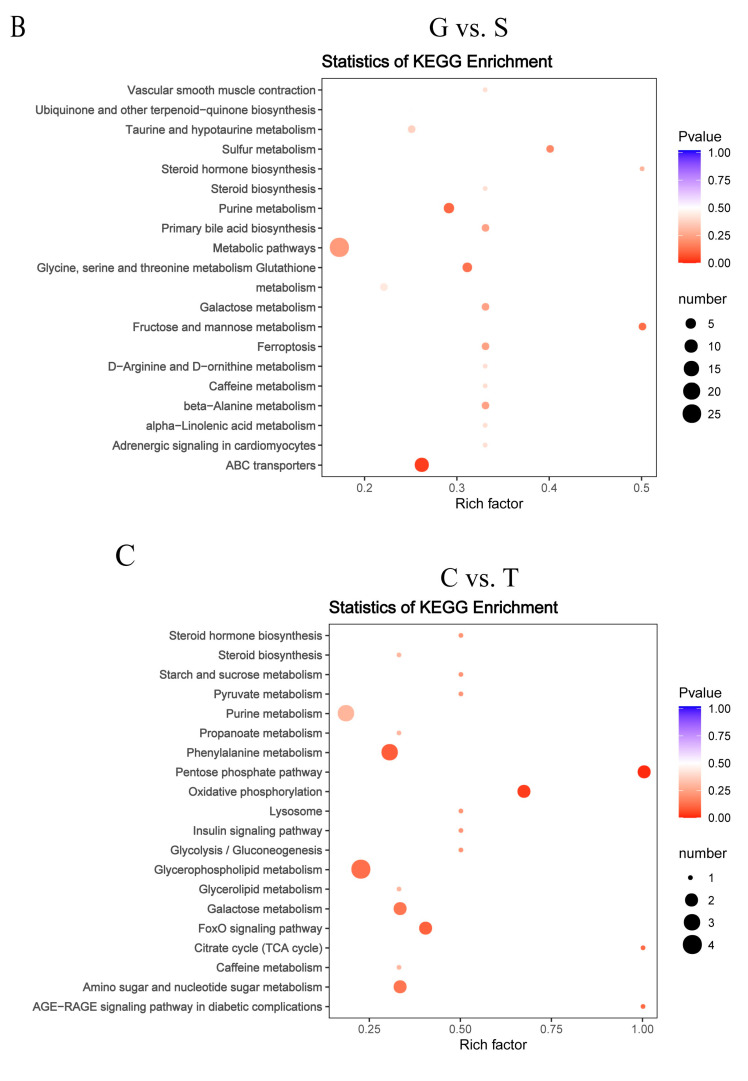

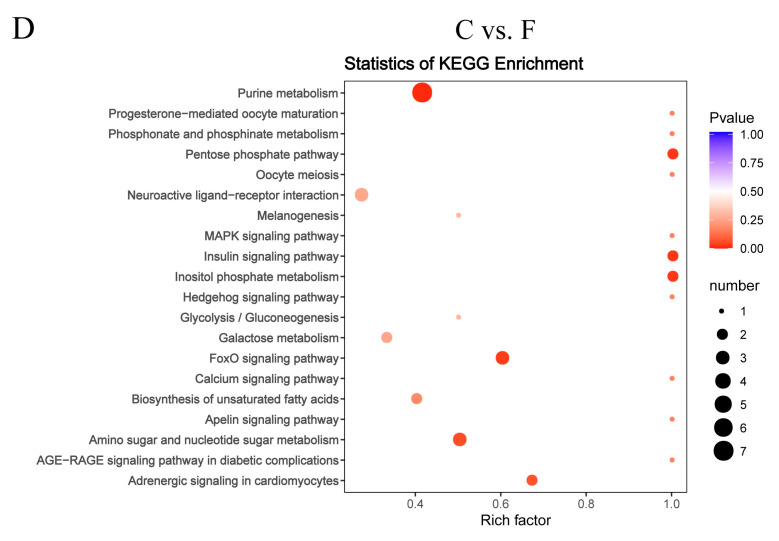
Topology analysis of metabolic pathways identified in the GCs of goose overexpressed or knockdown SCD. N vs. S comparison (**A**), G vs. S comparison (**B**), C vs. T comparison (**C**), and C vs. F comparison (**D**). Advanced bubble chart shows the enrichment of differentially abundant metabolites in pathways. The *x*-axis represents the rich factor (rich factor = number of different metabolites enriched in the pathway/number of all metabolites in the background metabolites set). The *y*-axis represents the enriched pathways. Size of the bubble represents the number of different abundant metabolites enriched in the pathway, and the color represents enrichment significance.

A functional analysis of pathways related to the differential abundances of metabolites was conducted using KEGG analysis. As shown in Supplementary Material Table S3, 46 of these differentially expressed metabolites were associated with 58 metabolic pathways in the N vs. S comparison, 31 metabolites with 40 metabolic pathways in G vs. S comparison, 22 metabolites with 38 metabolic pathways in C vs. T comparison, and 32 metabolites with 59 metabolic pathways in C vs. F comparison. We performed topology-based pathway analysis of the top 20 pathways found in the overexpressing and knockdown groups. Figure 7 shows that SCD overexpression had a significant effect on taurine and hypotaurine metabolism; sulfur metabolism; primary bile acid biosynthesis; glycine, serine, and threonine metabolism; glutathione metabolism; and ABC transporters. The knockdown of SCD had a significant effect on purine metabolism, the insulin signaling pathway, glycolysis/gluconeogenesis, galactose metabolism, the FoxO signaling pathway, amino sugar and nucleotide sugar metabolism, and the AGE-RAGE signaling pathway in diabetic complications. Both the overexpressed and knockdown groups were metabolically enriched in 10 lipid-related pathways (Table 2); the 25 other enriched lipid-related metabolic pathways of overexpressed or knockdown groups are shown in Table S4. Of these, tyrosine metabolism, arginine biosynthesis/D-arginine and D-ornithine metabolism, alpha-linolenic acid metabolism, and taurine and hypotaurine metabolism were enriched in the metabolic pathway that was specifically enriched in the overexpressed group. Glycolysis/gluconeogenesis, the insulin signaling pathway, and lysosome were enriched in the metabolic pathway that was specifically enriched in the knockdown group.

## 4. Discussion

The expression of SCD is tissue-specific and sex-dependent. Previous studies in chickens revealed that the second-highest *SCD* expression level occurs in the ovaries [42]. This could be due to differences in fat deposition and/or levels of hormones, particularly sex hormones, within tissues (e.g., estrogen, androgen, and GH) [43]. Our results showed that *SCD* expression was significantly higher in the granulosa than in the theca layer (Figure 1B). We therefore speculated that the function of *SCD* in goose GCs is closely related to follicular development. Understanding the mechanisms through which the processes associated with follicular recruitment and selection occur has great value for follicular development research [25]. In this study, we demonstrated that the expression levels of *SCD* increase during follicular recruitment and selection processes, reaching a peak during ovulation (Figure 1B). Many developing follicles undergo atresia as they advance toward ovulation, except for those undergoing the recruitment and selection processes. It is well known that GC death via apoptosis is the main cause of atresia [44]. Our results suggest the vital role that *SCD* plays in inhibiting GC apoptosis. Emerging evidence suggests a crucial role for *SCD* in the regulation of programmed cell death and lipid-mediated cytotoxicity [45]. The antiapoptotic effect of *SCD* can likely be attributed to the conversion of excess toxic SFAs into relatively harmless MUFAs by desaturase, which regulates the ratio of SFAs available for modulating membrane fluidity and signal transduction [46].

The intracellular concentration of SCD fluctuates widely in response to complex and often competing hormonal and dietary factors. A combination of transcriptional regulation and rapid protein degradation produces transient elevations in SCD activity in response to physiological demands [47]. We found that *SCD* was expressed in a time-dependent dynamic oscillation pattern during the seven days of primary in vitro GC culture; this trend was similar to that observed for SCD activity (Figure 2). The observation that changes in mRNA expression are paralleled by changes in the activity of the corresponding enzyme allows us to conclude that the change in enzyme activity is due to altered gene expression. Experiments on the liver support the idea that the *SCD* mRNA levels are directly indicative of the desaturase activity; a modification of SCD activity in liver cells can be achieved by modifying the *SCD* mRNA level [48]. This enzymatic reaction plays a critical role in directing the cell towards either lipid synthesis or oxidation and produces diverse effects on cellular function [49]. We hypothesize that SCD is a primary regulator of the process of lipid homeostasis in goose GCs. Studies using the CHO cell line confirm this view. SCD activity was shown to increase when CHO cells were incubated with lipid-depleted media; however, when the medium was supplemented with cholesterol, the desaturase activity was found to be reduced [50].

**Figure 8:**
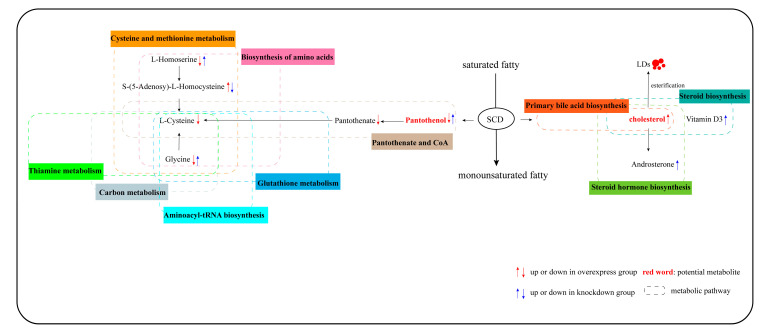
Potential metabolic pathway in goose GCs induced by overexpressed and knockdown SCD. Notes: “↑” and “↓” in red indicate metabolites which are upregulated in the overexpressed SCD group and are downregulated in the control group, respectively; “↑” and “↓” in blue indicate metabolites which are upregulated in the knockdown SCD group and downregulated in the control group. All MS/MS spectra of those important metabolites are shown in Figure S3.

This study revealed the metabolites and the metabolic pathways in SCD-overexpressing and SCD-knockdown GCs based on metabolomic analysis. Our study showed that 10 lipid-related pathways were enriched in overexpressed and knockdown SCD groups. Lipid-related functions are shown in Table 1. The results reported here provide new evidence that SCD functions in the regulation of lipid metabolic processes during goose follicle development. The overexpression of SCD enhanced the intracellular level of cholesterol, which is involved in the steroid hormone biosynthesis/steroid biosynthesis pathway (Table 1 and Figure S2). We speculate that the overexpression of SCD might facilitate the synthesis and subsequent esterification of cholesterol into lipid droplets (LDs) in goose GCs. Our recently published study demonstrated that vital miRNA–mRNA interactions related to lipid regulation, including LD formation, occur during goose follicular selection [19]. Furthermore, our research indicated that LD accumulation capacity depends on the stage of follicle development, with the highest lipid content found in F1 GCs [51]. The in vivo results of this study similarly showed peak SCD expression in F1 granulosa layers (Figure 1). These findings support the possibility that a similar mechanism transpires in mammals, in which lipid metabolism is assumed to be essential for follicular development and the oocyte energy supply [52,53]. In addition, while the knockdown of SCD maximally enhanced the content of pantothenol, which is a component of pantothenate and coenzyme A (CoA), SCD overexpression decreased the levels of pantothenol and pantothenate (Table 1 and Figure S2). Based on this finding, we postulate for the first time that pantothenate and CoA participate in goose follicle lipid metabolism. Pantothenate forms the core of CoA, which occurs in sequestered pools in eukaryotic cells and plays important roles in lipid synthesis, microsomal fatty acid oxidation, protein modifications, and membrane trafficking [54,55]. Cholesterol and pantothenol or pantothenate could potentially serve as metabolite biomarkers; the pathways underlying these associations are shown in Figure 8. An important avenue of future study entails research into the potential functions of SCD in lipid metabolism in goose GCs.

## 5. Conclusions

Our findings point to the vital role of SCD during follicular development, based on a comparison of SCD-overexpressing cells with SCD-knockdown cells, detailing the lipid metabolism-related pathways that are affected by both of these conditions. Moreover, we confirmed cholesterol and pantothenol/pantothenate as potential metabolite biomarkers for research on lipid metabolism by SCD in goose GCs. These understandings should shed light on the mechanisms underlying lipid metabolism during avian follicular development.

## Figures and Tables

**Table 1:** Details of the sequences of siRNAs and primers for GFP-SCD.

Name	Sense Sequence (5′-3′)	Antisense Sequence (5′-3′)
siRNA210	GCGAUACGUCUGGAGGAAUTT	AUUCCUCCAGACGUAUCGCTT
siRNA405	GCGGAUCUUCUUGACUAUUTT	AAUAGUCAAGAAGAUCCGCTT
siRNA774	GCUCAACGCCACUUGGCUATT	UAGCCAAGUGGCGUUGAGCTT
siRNA-scrambled	UUCUCCGAACGUGUCACGUTT	ACGUGACACGUUCGGAGAATT
primers for GFP-SCD	CCGCTCGAGATGGAGAAGGACTTACTCAGTCATG	CCCAAGCTTTCAGCCGCTCTTGTGACTCCC

**Table 2:** Metabolic pathways and significantly different metabolites associated with lipid metabolism and enriched in both the overexpressed and the knockdown groups.

Pathway	N vs. S	G vs. S	C vs. T	C vs. F	Lipid-Related Functions Reported	Reference
Steroid hormone biosynthesis/Steroid biosynthesis	Cholesterol	Cholesterol	Androsterone/Vitamin D3	Androsterone	Steroid hormones are essential regulators of a vast number of physiological processes.	[31]
Galactose metabolism	UDP-glucose	D-Sorbitol; Dulcitol	D-Glucose; D-Sorbitol	D-Glucose; Myoinositol	Galactose exerts primarily suppressive effects of ovarian follicle number and steroid secretion by direct actions on the ovary.	[32]
Tryptophan metabolism	N-Acetyl-5-Hydroxytryptamine; Anthranilic acid	N-Acetyl-5-Hydroxytryptamine	Succinic Acid	Epinephrine	Molecular modeling studies suggested favorable stacking interactions between cholesterol and tryptophan, in which the face of the complex ring system of cholesterol and the indole ring of tryptophan build the interaction interface.	[33]
Sulfur metabolism	L-Cysteine; 2-Aminoethanesulfonic Acid	2-Aminoethanesulfonic Acid; L-Homoserine	Succinic Acid	L-Homoserine	Recently, increasing attention has been paid to the role of sulfur amino acids in regulating lipid metabolism.	[34]
Cysteine and methionine metabolism	L-Cysteine; S-Sulfo-L-Cysteine; L-Alanine; Glutathione Reducedform; S-(5-Adenosy)-L-Homocysteine	L-Homoserine; L-Cystine	S-Sulfo-L-Cysteine	L-Homoserine; S-(5-Adenosy)-L-Homocysteine	Much attention has been recently focused on the effects of methionine restriction and cysteine on metabolic health, especially lipid metabolism.	[35,36]
pyrimidine metabolism	Uridine 5-Monophosphate; UDP-glucose	Β-Pseudouridine	Cytidine; 2-Deoxyribose 1-Phosphate	2-Deoxyribose 1-Phosphate	Given the link between pyrimidine metabolism and liver lipid accumulation, there is a potential for the use of nucleosides and nucleoside analogs in the treatment of fatty liver conditions.	[37]
Pantothenate and CoA biosynthesis	Pantothenate; L-Cysteine	Pantothenol	Pantothenol	Pantothenol	Pantothenate forms the core of CoA and is a precursor to acyl carrier protein (ACP), making it essential in both energy and lipidmetabolism.	[38]
Biosynthesis of amino acids	Anthranilic acid; Glycine; S-Sulfo-L-Cysteine; S-(5-Adenosy)-L-Homocysteine; L-Threonine; L-Alanine; L-Cysteine; L-Ornithine; L-Isoleucine	L-Ornithine; L-Homoserine; L-Threonine; Shikimic Acid	S-Sulfo-L-Cysteine	L-Homoserine; Glycine; L-Asparagine Anhydrous; S-(5-Adenosy)-L-Homocysteine	Amino acids were distributed in a lipid bilayer.	[39]
ABC transporters	2-Aminoethanesulfonic Acid; Spermidine; proline betaine; Glycine; Betaine; Glutathione Reducedform; L-Ornithine; L-Isoleucine; Biotin; Choline; L-Threonine; L-Alanine; Inosine	Betaine; D-Sorbitol; Inosine; L-Cystine; L-Threonine; L-Ornithine; D-Mannitol; Spermidine; Guanosine; proline betaine; 2-Aminoethanesulfonic Acid; Xanthosine	D-Sorbitol; Xanthosine; Glycerol 3-phosphate; D-Glucose; Cytidine	Biotin; Myoinositol; Spermidine; Glycerol 3-phosphate; Glycine; Inosine; Guanosine; D-Glucose	ATP binding cassette (ABC) transporter proteins are thought to facilitate the ATP-dependent translocation of lipids or lipid-related compounds—such substrates include cholesterol, plant sterols, bile acids, phospholipids and sphingolipids.	[40]
Neuroactive ligand-receptor interaction	2-Aminoethanesulfonic Acid; L-Thyroxine; Glycine	2-Aminoethanesulfonic Acid; Norepinephrine	Adenosine 5′-Diphosphate	Adenosine 5′-Diphosphate; Epinephrine; Glycine	Modulation of neurotransmitter receptors by lipids occurs at multiple levels, affecting a wide variety of activities, including their trafficking, sorting, stability, residence lifetime at the cell surface, endocytosis, and recycling, among other important functional properties at the synapse.	[41]

siRNA: small interfering RNA.

## Supplementary Materials

The following are available online at https://www.mdpi.com/2073-4425/11/9/1001/s1, Figure S1. Unsupervised hierarchical clustering of all metabolites in each group; each column denotes one group. Increased metabolite concentrations are shown in red, whereas decreased metabolite concentrations are shown in blue. Figure S2. Top 20 different metabolites according to the log2^FC^ in N vs. S comparison (A), G vs. S comparison (B), C vs. T comparison (C), and C vs. F comparison (D). Figure S3. MS/MS spectrum for the most important metabolites. Androsterone (A), cholesterol (B), glycine (C), L-cysteine (D), L-homoserine (E), pantothenate (F), pantothenol (G), S-(5-adenosy)-L-homocysteine (H) and vitamin D3 (I). Table S1. Details of the primers used for quantitative real-time PCR analysis. Table S2. Significant differential metabolites involved in N vs. S comparison, G vs. S comparison, C vs. T comparison, and C vs. F comparison. Table S3. A functional analysis of pathways related to the differentially abundant metabolites. Table S4. Metabolic pathways and significantly different metabolites are associated with lipid metabolism and enriched in the overexpressed or knockdown groups.

## Abbreviations

CoA	coenzyme A
GC	granulosa cell
KEGG	Kyoto Encyclopedia of Genes and Genomes
LIT	linear ion trap
LC-ESI-MS/MS	liquid chromatography-electrospray ionization-tandem mass spectrometry
LC-MS/MS	liquid chromatography-tandem mass spectrometry
MUFA	monounsaturated fatty acid
MRM	multiple reaction monitoring
OPLS-DA	orthogonal correction partial least squares discriminant analysis
PUFA	polyunsaturated fatty acid
PCA	principal component analysis
QC	quality control
qRT-PCR	quantitative real-time PCR
SFA	saturated fatty acid
siRNA	small interfering RNA
SCD	stearoyl-CoA desaturase
QQQ	triple quadrupole
VIP	variable importance in projection
LD	lipid droplet
