# Supplementary material for: Metabolomic Analysis of SCD during Goose Follicular Development: Implications for Lipid Metabolism
Source: Genes (Basel). 2020 Aug 26;11(9):1001. doi: 10.3390/genes11091001 (PMC7565484; doi:10.3390/genes11091001)
Supplement: Supplementary file 1 [file genes-11-01001-s001.zip › Supplementary data/Table S 1.docx]

**S. Table 1 Details of the primers used for quantitative real-time PCR analysis**

| Gene | primer sequences | accession number | amplicon size (bp) |
| --- | --- | --- | --- |
| SCD | Forward: GCCATCGGTCCTACAAAGC  Reverse: AGCCAATGTGGGAGAAGAAA | XM_013201691 | 180 |
| β-actin | Forward:CAACGAGCGGTTCAGGTGT  Reverse:TGGAGTTGAAGGTGGTCTCG | M26111.1 | 92 |
| GAPDH | Forward: TTTCCCCACAGCCTTAGCA  Reverse: GCCATCACAGCCACACAGA | MG674174.1 | 86 |

Notes: β-actin and ribosomal GAPDH were selected as the reference genes
