# Supplementary material for: Metabolomic Analysis of SCD during Goose Follicular Development: Implications for Lipid Metabolism
Source: Genes (Basel). 2020 Aug 26;11(9):1001. doi: 10.3390/genes11091001 (PMC7565484; doi:10.3390/genes11091001)
Supplement: Supplementary file 1 [file genes-11-01001-s001.zip › Supplementary data/Table S 2.docx]

**Table S2. Significant differential metabolites involved in** **N vs. S comparison, G vs. S comparison, C vs. T comparison, and C vs. F comparison.**

| experimental vs. control | Compounds | Class | \| VIP \| \| --- \| | Fold Change |
| --- | --- | --- | --- | --- | --- |
| N vs. S comparison | L-Threonine | Amino Acid metabolomics | 1.166581 | 0.770575 |
|  | L-Isoleucine | Amino Acid metabolomics | 1.595504 | 0.751636 |
|  | L-Cysteine | Amino Acid metabolomics | 1.006051 | 1.270395 |
|  | Uridine 5'-Diphospho-N-Acetylgalactosamine | Nucleotide metabolomics | 1.926345 | 0.645292 |
|  | D-Arabinose | Carbohydrate metabolomics | 1.275618 | 0.669456 |
|  | Pantothenate | CoOthersEnzyme Factor & vitamin | 1.871372 | 1.651484 |
|  | 2-Aminoethanesulfinic Acid | Organic Acid And Its Derivatives | 1.364089 | 2.432057 |
|  | 4-Guanidinobutyric Acid | Organic Acid And Its Derivatives | 1.635071 | 1.358729 |
|  | Guanidinoethyl Sulfonate | Organic Acid And Its Derivatives | 1.656407 | 1.456403 |
|  | Lysope 18:1 | LipidsOthersPhospholipid | 2.058041 | 0.598281 |
|  | Lysope 14:0 | LipidsOthersPhospholipid | 1.602133 | 0.71861 |
|  | Palmitoleic Acid (C16:1) | Lipids Fatty Acids | 1.443164 | 0.807696 |
|  | Hexadecanoic Acid (C16:0) | Lipids Fatty Acids | 2.037213 | 0.779778 |
|  | Linoleic Acid (C18:2N6C) | Lipids Fatty Acids | 1.503776 | 0.740671 |
|  | EPA [5Z,8Z,11Z,14Z,17Z-eicosapentaenoic acid] | Oxidized lipid | 2.121368 | 0.698998 |
|  | Dl-Glyceraldehyde3-Phosphate | Organic Acid And Its Derivatives | 1.208903 | 1.460089 |
|  | 8,15-Dihete | Lipids Fatty Acids | 1.472742 | 0.753298 |
|  | ADP-ribose | Nucleotide metabolomics | 1.446319 | 0.56141 |
|  | UDP-glucose | Nucleotide metabolomics | 1.108433 | 1.348523 |
|  | 3'-Sialyllactose | Carbohydrate metabolomics | 1.176497 | 1.393958 |
|  | Anthranilic acid | Benzene and substituted derivatives | 1.119129 | 1.240463 |
|  | 2-n-Pentylfuran | Heterocyclic compound | 2.077512 | 1.696288 |
|  | PHENYL-BETA-D-GLUCOPYRANOSIDE | Carbohydrate metabolomics | 1.44533 | 0.826652 |
|  | 11,12-EET [(±)11,(12)-epoxy-5Z,8Z,14Z-eicosatrienoic acid] | Oxidized lipid | 1.683255 | 0.60171 |
|  | Xylose | Carbohydrate metabolomics | 1.282173 | 0.61656 |
|  | Glycine | Amino Acid metabolomics | 1.249167 | 1.347418 |
|  | L-Ornithine | Amino Acid metabolomics | 1.002522 | 1.232776 |
|  | L-Alanine | Amino Acid metabolomics | 1.376997 | 1.281004 |
|  | 5-Oxoproline | Amino Acid metabolomics | 1.152421 | 1.216228 |
|  | Betaine | Alkaloid | 1.705081 | 1.280789 |
|  | Glutathione Reducedform | Amino Acid metabolomics | 1.500861 | 1.218451 |
|  | N-Acetylmannosamine | Amino Acid metabolomics | 1.452555 | 0.761127 |
|  | Phe-Phe | Amino Acid metabolomics | 1.374605 | 1.657823 |
|  | S-(5-Adenosy)-L-Homocysteine | Amino Acid metabolomics | 1.570841 | 0.779227 |
|  | S-Sulfo-L-Cysteine | Amino Acid metabolomics | 1.4421 | 1.228049 |
|  | Choline | Cholines | 1.942624 | 1.250325 |
|  | Biotin | CoOthersEnzyme Factor & vitamin | 2.052972 | 0.524219 |
|  | 1-Methylhistidine | Amino Acid metabolomics | 1.485412 | 0.749267 |
|  | 2'-Deoxyadenosine-5'-Monophosphate | Nucleotide metabolomics | 1.270204 | 1.219186 |
|  | Inosine | Nucleotide metabolomics | 1.2534 | 0.716717 |
|  | Nicotinic Acid Adenine Dinucleotide | Nucleotide metabolomics | 1.951174 | 1.489939 |
|  | L-Thyroxine | Hormones | 1.80818 | 1.320114 |
|  | L-Carnitine | Camitine | 1.018301 | 1.476332 |
|  | N-Acetyl-5-Hydroxytryptamine | Tryptamines And Its Derivatives | 1.342633 | 1.393224 |
|  | Nicotinamide | CoOthersEnzyme Factor & vitamin | 2.207492 | 0.625963 |
|  | 2-Aminoethanesulfonic Acid | Organic Acid And Its Derivatives | 1.498866 | 1.83333 |
|  | Lysopc 14:0 | LipidsOthersPhospholipid | 1.466986 | 0.790346 |
|  | Lysopc 18:1 | LipidsOthersPhospholipid | 1.505233 | 0.818472 |
|  | Lysopc 18:2 | LipidsOthersPhospholipid | 1.649611 | 0.730085 |
|  | Uridine 5-Monophosphate | Nucleotide metabolomics | 1.232387 | 1.241908 |
|  | 2-(Dimethylamino)Guanosine | Nucleotide metabolomics | 1.882749 | 1.266323 |
|  | Hypoxanthine-9-β-D-Arabinofuranoside | Nucleotide metabolomics | 1.279246 | 0.703484 |
|  | 3-Aminoisobutanoic Acid | Amino Acid metabolomics | 1.063769 | 1.232797 |
|  | Cholesterol | Lipids | 1.034994 | 0.40025 |
|  | Acetyl-L-carnitine | Camitine | 1.639489 | 1.872042 |
|  | DL-Carnitine | Camitine | 1.265174 | 1.395191 |
|  | Isobutyryl carnitine | Camitine | 1.415856 | 1.202277 |
|  | Stearidonic Acid | Lipids Fatty Acids | 1.576561 | 0.580342 |
|  | N-Acetylphenylalanine | Organic Acid And Its Derivatives | 1.275144 | 1.238213 |
|  | p-Mentha-1,3,8-triene | Lipids Fatty Acids | 1.4553 | 0.582144 |
|  | 2'-Hydroxy-5'-methylacetophenone | Benzene and substituted derivatives | 1.655234 | 0.774503 |
|  | Spermidine | Polyamine | 1.165594 | 1.773585 |
|  | 2-Methylbutyroylcarnitine | Lipids Fatty Acids | 1.904046 | 1.500747 |
|  | Isonicotinic acid | Organic Acid And Its Derivatives | 2.199563 | 0.605853 |
|  | Butylamine | Amines | 1.564929 | 0.830504 |
|  | Hexyl butyrate | Fatty acyls | 1.062761 | 1.248775 |
|  | 9-Fluorenone | Ketones | 1.636177 | 0.767032 |
|  | Aspirin | Organic Acid And Its Derivatives | 1.465994 | 1.278812 |
|  | Octanal | Aldehyde | 1.206962 | 0.643862 |
|  | 6-Methylnicotinamide | Pyridine And Pyridine Derivatives | 1.255745 | 0.725056 |
|  | FURFURYL ALCOHOL | Alcohol | 1.056649 | 1.345687 |
|  | Choline chloride | Others | 1.803016 | 1.263389 |
|  | proline betaine | Organic Acid And Its Derivatives | 1.553355 | 1.224865 |
|  | Phosphocholine | Nucleotide metabolomics | 1.523384 | 1.347726 |
|  | 2,4-Dihydroxypteridine | Pteridines and derivatives | 1.586712 | 1.275885 |
| G vs. S comparison | L-Cystine | Amino Acid metabolomics | 1.066191 | 0.769272 |
|  | L-Threonine | Amino Acid metabolomics | 1.056487 | 0.820024 |
|  | Dulcitol | Carbohydrate metabolomics | 1.397582 | 0.477443 |
|  | Adenosine 5'-Monophosphate | Nucleotide metabolomics | 1.236762 | 1.284288 |
|  | Guanosine | Nucleotide metabolomics | 1.747218 | 0.734572 |
|  | D-Sorbitol | Carbohydrate metabolomics | 1.290252 | 0.509745 |
|  | Guanidinoethyl Sulfonate | Organic Acid And Its Derivatives | 1.019794 | 1.233168 |
|  | L-Homoserine | Organic Acid And Its Derivatives | 1.368344 | 1.212892 |
|  | Shikimic Acid | Organic Acid And Its Derivatives | 1.33154 | 0.803011 |
|  | Β-Pseudouridine | Nucleotide metabolomics | 1.385199 | 1.261014 |
|  | D-Sedoheptuiose 7-Phosphate | Carbohydrate metabolomics | 1.054506 | 1.21355 |
|  | Xanthosine | Nucleotide metabolomics | 1.859152 | 0.736306 |
|  | DL-Benzylsuccinic acid | Lipids Fatty Acids | 1.835005 | 0.513761 |
|  | Gamma-Glu-Leu | Organic Acid And Its Derivatives | 1.432343 | 0.720188 |
|  | 2-n-Pentylfuran | Heterocyclic compound | 1.797143 | 1.277766 |
|  | Propylparaben | Benzoic Acid And Its Derivatives | 2.40143 | 0.72855 |
|  | TxB3 [9α,11,15S-trihydroxythromba-5Z,13E,17Z-trien-1-oic acid] | Oxidized lipid | 1.194609 | 1.250013 |
|  | L-Ornithine | Amino Acid metabolomics | 1.212506 | 1.223482 |
|  | Betaine | Alkaloid | 1.740578 | 1.268644 |
|  | L-Carnosine | Amino Acid metabolomics | 1.646091 | 0.683404 |
|  | Phe-Phe | Amino Acid metabolomics | 1.863352 | 0.829998 |
|  | 2-Hydroxy-6-Aminopurine | Nucleotide metabolomics | 1.626917 | 0.795382 |
|  | 5,6-Dihydro-5-Methyluracil | Nucleotide metabolomics | 2.426315 | 0.645438 |
|  | Adenine | Nucleotide metabolomics | 1.703484 | 0.832136 |
|  | Inosine | Nucleotide metabolomics | 1.490765 | 0.747616 |
|  | Norepinephrine | Hormones | 1.37947 | 1.404965 |
|  | N-Acetyl-5-Hydroxytryptamine | Tryptamines And Its Derivatives | 1.069971 | 0.71911 |
|  | D-Mannitol | Carbohydrate metabolomics | 1.404002 | 0.737354 |
|  | Pantothenol | CoOthersEnzyme Factor & vitamin | 1.440058 | 3.874286 |
|  | 2-Aminoethanesulfonic Acid | Organic Acid And Its Derivatives | 1.356643 | 0.758363 |
|  | Adipic Acid | Organic Acid And Its Derivatives | 1.031219 | 0.812792 |
|  | D-Pipecolinic Acid | Organic Acid And Its Derivatives | 1.03222 | 0.745055 |
|  | 2-(Dimethylamino)Guanosine | Nucleotide metabolomics | 1.783296 | 0.745087 |
|  | H-Homoarg-Oh | Amino Acid metabolomics | 1.685 | 1.360554 |
|  | Hypoxanthine-9-β-D-Arabinofuranoside | Nucleotide metabolomics | 1.675856 | 0.717224 |
|  | Cholesterol | Lipids | 1.741346 | 0.220794 |
|  | Acetyl-L-carnitine | Camitine | 1.428027 | 1.402555 |
|  | Stearidonic Acid | Lipids Fatty Acids | 1.741784 | 0.597553 |
|  | p-Mentha-1,3,8-triene | Lipids Fatty Acids | 1.777454 | 0.565466 |
|  | 2'-Hydroxy-5'-methylacetophenone | Benzene and substituted derivatives | 1.72247 | 0.770917 |
|  | Spermidine | Polyamine | 1.196583 | 0.771723 |
|  | Triethylamine | Hydrocarbon derivative | 1.747962 | 1.629726 |
|  | o-Xylene | Benzene and substituted derivatives | 1.463955 | 0.819373 |
|  | 9-Fluorenone | Ketones | 1.789547 | 0.749386 |
|  | Aspirin | Organic Acid And Its Derivatives | 1.591425 | 1.376088 |
|  | Octanal | Aldehyde | 1.028795 | 0.71239 |
|  | 6-Methylnicotinamide | Pyridine And Pyridine Derivatives | 1.715487 | 0.742674 |
|  | proline betaine | Organic Acid And Its Derivatives | 1.570476 | 1.228874 |
|  | Vitamin E | CoOthersEnzyme Factor & vitamin | 1.869686 | 1.201491 |
|  | Oleate | Lipids | 1.104677 | 0.677803 |
| C vs. T comparison | Benzoic Acid | Benzene and substituted derivatives | 2.617169 | 0.792798 |
|  | 5-Hydroxymethyluracil | Nucleotide metabolomics | 1.81903 | 0.767052 |
|  | Succinic Acid | Amino Acid metabolomics | 1.467556 | 1.272111 |
|  | D-Sorbitol | Carbohydrate metabolomics | 1.189368 | 0.53977 |
|  | D-Glucose | Carbohydrate metabolomics | 1.251037 | 0.599055 |
|  | Vitamin D3 | CoOthersEnzyme Factor & vitamin | 1.956893 | 0.801948 |
|  | Lysope 14:0 | LipidsOthersPhospholipid | 1.724609 | 0.796675 |
|  | Dl-Glyceraldehyde3-Phosphate | Organic Acid And Its Derivatives | 1.893671 | 0.706925 |
|  | N-Acetylmethionine | Amino Acid metabolomics | 1.504602 | 0.759942 |
|  | 2-Deoxyribose 1-Phosphate | Carbohydrate metabolomics | 1.175215 | 1.316544 |
|  | Xanthosine | Nucleotide metabolomics | 1.454235 | 0.743379 |
|  | 3'-Sialyllactose | Carbohydrate metabolomics | 1.399111 | 0.570141 |
|  | Glycerol 3-phosphate | Lipids Fatty Acids | 1.926232 | 0.662878 |
|  | Ethylsalicylate | Organic Acid And Its Derivatives | 2.242475 | 0.785147 |
|  | PHENYL-BETA-D-GLUCOPYRANOSIDE | Carbohydrate metabolomics | 2.122599 | 0.733233 |
|  | L-Homocitrulline | Amino Acid metabolomics | 1.386757 | 0.552284 |
|  | N-Acetylmannosamine | Amino Acid metabolomics | 1.70513 | 0.761648 |
|  | S-Sulfo-L-Cysteine | Amino Acid metabolomics | 1.56886 | 1.433548 |
|  | 1-Methylhistidine | Amino Acid metabolomics | 2.048573 | 0.68965 |
|  | Adenine | Nucleotide metabolomics | 1.377542 | 1.335071 |
|  | Adenosine 5'-Diphosphate | Nucleotide metabolomics | 1.110245 | 1.224363 |
|  | Cytidine | Nucleotide metabolomics | 1.412542 | 0.773778 |
|  | L-Carnitine | Camitine | 1.063465 | 1.300756 |
|  | Pantothenol | CoOthersEnzyme Factor & vitamin | 2.35502 | 0.329005 |
|  | Adipic Acid | Organic Acid And Its Derivatives | 2.023728 | 0.690829 |
|  | D-Pipecolinic Acid | Organic Acid And Its Derivatives | 1.56952 | 0.688934 |
|  | Lysopc 14:0 | LipidsOthersPhospholipid | 2.195384 | 0.813178 |
|  | Lysopc 18:1 | LipidsOthersPhospholipid | 1.831296 | 0.823025 |
|  | 2-(Dimethylamino)Guanosine | Nucleotide metabolomics | 1.746555 | 1.238581 |
|  | 3-Aminoisobutanoic Acid | Amino Acid metabolomics | 1.647882 | 1.219355 |
|  | Androsterone | Hormones | 1.130701 | 0.745289 |
|  | N-Acetylphenylalanine | Organic Acid AndIts Derivatives | 1.19715 | 1.210006 |
|  | Dimethyl fumarate | Lipids Fatty Acids | 1.553017 | 1.309987 |
|  | Aspirin | Organic Acid And Its Derivatives | 1.280605 | 1.247804 |
|  | 1-Phenylethanol | Benzene and substituted derivatives | 1.896614 | 1.321415 |
|  | N-Methyl-L-Glutamate | Amino Acid metabolomics | 1.001583 | 1.280792 |
|  | Phosphocholine | Nucleotide metabolomics | 1.414015 | 1.208085 |
| C vs. F comparison | L-Asparagine Anhydrous | Amino Acid metabolomics | 1.044559 | 0.82951 |
|  | Inositol | Carbohydrate metabolomics | 1.165345 | 0.712706 |
|  | Adenosine 5'-Monophosphate | Nucleotide metabolomics | 2.310015 | 1.442028 |
|  | Guanosine | Nucleotide metabolomics | 1.090902 | 0.757455 |
|  | D-Glucose | Carbohydrate metabolomics | 1.568367 | 0.743132 |
|  | D-Erythronolactone | Carbohydrate metabolomics | 1.203815 | 1.769688 |
|  | D-Arabinose | Carbohydrate metabolomics | 1.747046 | 0.799768 |
|  | L-Homoserine | Organic Acid And Its Derivatives | 2.093097 | 0.760578 |
|  | N'-Formylkynurenine | Organic Acid And Its Derivatives | 1.785829 | 1.211613 |
|  | Lysope 14:0 | LipidsOthersPhospholipid | 1.181047 | 0.831047 |
|  | Palmitoleic Acid (C16:1) | Lipids Fatty Acids | 1.703514 | 0.727405 |
|  | Linoleic Acid (C18:2N6C) | Lipids Fatty Acids | 1.424731 | 0.792725 |
|  | EPA [5Z,8Z,11Z,14Z,17Z-eicosapentaenoic acid] | Oxidized lipid | 1.310543 | 0.759074 |
|  | Cyclic Amp | Nucleotide metabolomics | 1.022176 | 1.201675 |
|  | 8,15-Dihete | Lipids Fatty Acids | 1.519958 | 0.797899 |
|  | N-Acetylmethionine | Amino Acid metabolomics | 1.509112 | 0.663544 |
|  | 2-Deoxyribose 1-Phosphate | Carbohydrate metabolomics | 1.515328 | 1.518962 |
|  | 2-Methylbenzoic acid | Benzene and substituted derivatives | 1.251075 | 0.831077 |
|  | Glycerol 3-phosphate | Lipids Fatty Acids | 1.934308 | 0.820128 |
|  | Hexadecanedioic acid | Lipids Fatty Acids | 1.132609 | 0.817291 |
|  | Ethylsalicylate | Organic Acid And Its Derivatives | 2.04064 | 0.818804 |
|  | 2-n-Pentylfuran | Heterocyclic compound | 1.580451 | 1.512083 |
|  | N-(2-Methylbenzoyl)glycine | Amino Acid metabolomics | 1.694821 | 1.364643 |
|  | Sorbic acid | Organic Acid And Its Derivatives | 1.255515 | 0.758292 |
|  | Scyllo inositol | Alcohol | 1.281458 | 0.694204 |
|  | Glycine | Amino Acid metabolomics | 1.356118 | 0.762913 |
|  | 3-Chloro-L-Tyrosine | Amino Acid metabolomics | 2.127306 | 1.380114 |
|  | 5-Oxoproline | Amino Acid metabolomics | 1.532279 | 0.806882 |
|  | Methionine Sulfoxide | Amino Acid metabolomics | 1.239271 | 1.220714 |
|  | N-Acetylmannosamine | Amino Acid metabolomics | 1.797023 | 0.747922 |
|  | S-(5-Adenosy)-L-Homocysteine | Amino Acid metabolomics | 1.119287 | 1.200706 |
|  | Diethanolamine | Polyamine | 1.045913 | 0.737731 |
|  | Myoinositol | Carbohydrate metabolomics | 1.496871 | 1.220897 |
|  | Biotin | CoOthersEnzyme Factor & vitamin | 1.721203 | 1.250189 |
|  | Adenine | Nucleotide metabolomics | 1.088712 | 1.313726 |
|  | Adenosine 5'-Diphosphate | Nucleotide metabolomics | 1.054579 | 1.239463 |
|  | Inosine | Nucleotide metabolomics | 1.050419 | 0.693866 |
|  | Epinephrine | Hormones | 1.208291 | 0.624053 |
|  | Pantothenol | CoOthersEnzyme Factor & vitamin | 1.609643 | 0.128883 |
|  | Dodecanedioic Aicd | Organic Acid And Its Derivatives | 1.978535 | 0.685668 |
|  | 3-Aminoisobutanoic Acid | Amino Acid metabolomics | 1.728892 | 1.246718 |
|  | Glucosamine | Carbohydrate metabolomics | 1.481402 | 1.401939 |
|  | Androsterone | Hormones | 1.102852 | 0.804567 |
|  | Isobutyryl carnitine | Camitine | 1.77275 | 1.228316 |
|  | Spermidine | Polyamine | 1.555622 | 1.572525 |
|  | Dimethyl fumarate | Lipids Fatty Acids | 1.339396 | 1.272698 |
|  | Furfural | Organic Acid And Its Derivatives | 1.254706 | 1.356648 |
|  | Propylpropionate | Fatty acyls | 1.480719 | 0.750064 |
|  | m-Cresol | Phenols And Its Derivatives | 1.253022 | 1.303117 |
|  | 2,6-Di-tert-butyl-4-methylphenol | Benzene and substituted derivatives | 1.620179 | 1.495183 |
|  | 10-UNDECENOIC ACID | Fatty acyls | 1.782636 | 1.311227 |
|  | Octanal | Aldehyde | 1.73593 | 0.707398 |
|  | METHYL VALERATE | Fatty acyls | 1.279675 | 0.755882 |
|  | 6-Methylnicotinamide | Pyridine And Pyridine Derivatives | 1.026584 | 0.713472 |
|  | 3-Methylsalicylic acid | Organic Acid And Its Derivatives | 1.130442 | 0.830865 |
|  | 1-Phenylethanol | Benzene and substituted derivatives | 1.503887 | 1.255606 |
|  | Vitamin E | CoOthersEnzyme Factor & vitamin | 1.400357 | 0.784567 |
|  | Thiamine Monophosphate | Heterocyclic compound | 1.143919 | 0.760839 |

Defined as having a VIP ＞ 1 and fold-change ＞ 1.2
