# Supplementary material for: Metabolomic Analysis of SCD during Goose Follicular Development: Implications for Lipid Metabolism
Source: Genes (Basel). 2020 Aug 26;11(9):1001. doi: 10.3390/genes11091001 (PMC7565484; doi:10.3390/genes11091001)
Supplement: Supplementary file 1 [file genes-11-01001-s001.zip › Supplementary data/Table S 3.docx]

| **S. Table 3 A functional analysis of pathways related to the differentially abundant metabolites** | | | | |
| --- | --- | --- | --- | --- |
| Pathway | N vs. S comparison | G vs. S comparison | C vs. T comparison | C vs. F comparison |
| 2-Oxocarboxylic acid metabolism | L-Ornithine; L-Isoleucine | L-Ornithine | - | - |
| ABC transporters | 2-Aminoethanesulfonic Acid; Spermidine; proline betaine; Glycine; Betaine; Glutathione Reducedform; L-Ornithine; L-Isoleucine;Biotin; Choline; L-Threonine; L-Alanine; Inosine | Betaine; D-Sorbitol; Inosine; L-Cystine; L-Threonine; L-Ornithine; D-Mannitol; Spermidine; Guanosine; proline betaine; 2-Aminoethanesulfonic Acid; Xanthosine | D-Sorbitol; Xanthosine; Glycerol 3-phosphate; D-Glucose; Cytidine | Biotin; Myoinositol; Spermidine; Glycerol 3-phosphate; Glycine; Inosine; Guanosine; D-Glucose |
| Adrenergic signaling in cardiomyocytes | - | Norepinephrine | - | Epinephrine; Cyclic Amp |
| AGE-RAGE signaling pathway in diabetic complications | - | - | D-Glucose | D-Glucose |
| Alanine, aspartate and glutamate metabolism | L-Alanine | - | Succinic Acid | L-Asparagine Anhydrous |
| alpha-Linolenic acid metabolism | Stearidonic Acid | Stearidonic Acid | - | - |
| Amino sugar and nucleotide sugar metabolism | Uridine 5’-Diphospho-N-Acetylgalactosamine; N-Acetylmannosamine; UDP-glucose | - | D-Glucose; N-Acetylmannosamine | D-Glucose; N-Acetylmannosamine; Glucosamine |
| Aminoacyl-tRNA biosynthesis | L-Threonine; Glycine; L-Alanine; L-Cysteine; L-Isoleucine | L-Threonine | - | Glycine; L-Asparagine Anhydrous |
| Apelin signaling pathway | - | - | - | Cyclic Amp |
| Arginine and proline metabolism | 4-Guanidinobutyric Acid; L-Ornithine; Spermidine | L-Ornithine; Spermidine | - | Spermidine |
| Arginine biosynthesis | L-Ornithine | L-Ornithine | - | - |
| Ascorbate and aldarate metabolism | UDP-glucose | - | - | Myoinositol |
| beta-Alanine metabolism | Pantothenate; Spermidine | L-Carnosine; Spermidine | - | Spermidine |
| Biosynthesis of amino acids | Anthranilic acid; Glycine; S-Sulfo-L-Cysteine; S-(5-Adenosy)-L-Homocysteine; L-Threonine; L-Alanine; L-Cysteine; L-Ornithine; L-Isoleucine | L-Ornithine; L-Homoserine; L-Threonine; Shikimic Acid | S-Sulfo-L-Cysteine | L-Homoserine; Glycine; L-Asparagine Anhydrous; S-(5-Adenosy)-L-Homocysteine |
| Biosynthesis of unsaturated fatty acids | EPA [5Z,8Z,11Z,14Z,17Z-eicosapentaenoic acid]; Linoleic Acid (C18:2N6C); Hexadecanoic Acid (C16:0) | Oleate | - | EPA [5Z,8Z,11Z,14Z,17Z-eicosapentaenoic acid]; Linoleic Acid (C18:2N6C) |
| Biotin metabolism | Biotin | - | - | Biotin |
| Butanoate metabolism | - | - | Succinic Acid | - |
| Caffeine metabolism | - | Xanthosine | Xanthosine | - |
| Calcium signaling pathway | - | - | - | Cyclic Amp |
| Carbon metabolism | Glycine; L-Cysteine; L-Alanine | - | Succinic Acid | Glycine |
| Citrate cycle (TCA cycle) | - | - | Succinic Acid | - |
| Cysteine and methionine metabolism | L-Cysteine; S-Sulfo-L-Cysteine; L-Alanine; Glutathione Reducedform; S-(5-Adenosy)-L-Homocysteine | L-Homoserine; L-Cystine | S-Sulfo-L-Cysteine | L-Homoserine; S-(5-Adenosy)-L-Homocysteine |
| D-Arginine and D-ornithine metabolism | L-Ornithine | L-Ornithine | - | - |
| D-Glutamine and D-glutamate metabolism | 5-Oxoproline | - | - | 5-Oxoproline |
| Drug metabolism - other enzymes | Nicotinic Acid Adenine Dinucleotide; Isonicotinic acid | - | - | - |
| Fatty acid biosynthesis | Hexadecanoic Acid (C16:0); Palmitoleic Acid (C16:1) | Oleate | - | Palmitoleic Acid (C16:1) |
| Fatty acid degradation | Hexadecanoic Acid (C16:0) | - | - | - |
| Fatty acid elongation | Hexadecanoic Acid (C16:0) | - | - | - |
| Fatty acid metabolism | Hexadecanoic Acid (C16:0) | - | - | - |
| Ferroptosis | L-Cysteine; Glutathione Reducedform | L-Cystine; Vitamin E | - | Vitamin E |
| FoxO signaling pathway | - | Adenosine 5’-Monophosphate | Adenosine 5’-Diphosphate; D-Glucose | Adenosine 5’-Monophosphate; D-Glucose; Adenosine 5’-Diphosphate |
| Fructose and mannose metabolism | - | D-Mannitol; D-Sorbitol | D-Sorbitol | - |
| Galactose metabolism | UDP-glucose | D-Sorbitol; Dulcitol | D-Glucose; D-Sorbitol | D-Glucose; Myoinositol |
| Gap junction | - | Norepinephrine | - | Cyclic Amp |
| Glutathione metabolism | L-Ornithine; Spermidine; Glycine; L-Cysteine; Glutathione Reducedform | L-Ornithine; Spermidine | - | Glycine; Spermidine |
| Glycerolipid metabolism | UDP-glucose | - | Glycerol 3-phosphate | Glycerol 3-phosphate |
| Glycerophospholipid metabolism | Phosphocholine; Lysopc 14:0; Choline; Lysopc 18:2; Lysopc 18:1 | - | Lysopc 18:1; Phosphocholine; Lysopc 14:0; Glycerol 3-phosphate | Glycerol 3-phosphate; Diethanolamine |
| Glycine, serine and threonine metabolism | Betaine; Choline; Glycine; L-Threonine; L-Cysteine; proline betaine | L-Threonine; proline betaine; L-Homoserine; Betaine | - | L-Homoserine; Glycine |
| Glycolysis / Gluconeogenesis | - | - | D-Glucose | D-Glucose |
| Glyoxylate and dicarboxylate metabolism | Glycine | - | Succinic Acid | Glycine |
| GnRH signaling pathway | - | - | - | Cyclic Amp |
| Hedgehog signaling pathway | - | - | - | Cyclic Amp |
| Histidine metabolism | 1-Methylhistidine | L-Carnosine | 1-Methylhistidine | - |
| Linoleic acid metabolism | Linoleic Acid (C18:2N6C) | - | - | - |
| Inositol phosphate metabolism | - | - | - | Scyllo inositol; Myoinositol |
| Insulin signaling pathway | - | - | D-Glucose | Cyclic Amp; D-Glucose |
| Linoleic acid metabolism | - | - | - | Linoleic Acid (C18:2N6C) |
| Lysine degradation | Glycine | - | Succinic Acid | Glycine |
| Lysosome | - | - | Adenosine 5’-Diphosphate | Adenosine 5’-Diphosphate |
| MAPK signaling pathway | - | - | - | Cyclic Amp |
| Melanogenesis | - | - | - | Cyclic Amp |
| Metabolic pathways | 5-Oxoproline; Linoleic Acid (C18:2N6C); 9-Fluorenone; L-Alanine; Uridine 5-Monophosphate; 2’-Deoxyadenosine-5’-Monophosphate; N-Acetylmannosamine; Betaine;  4-Guanidinobutyric Acid; L-Isoleucine; 1-Methylhistidine; Choline  ; 2-Aminoethanesulfonic Acid; Spermidine; Hexadecanoic Acid (C16:0); Cholesterol; S-Sulfo-L-Cysteine; L-Ornithine; L-Thyroxine; Biotin; L-Threonine; Pantothenate; Inosine; S-(5-Adenosy)-L-Homocysteine; Uridine 5’-Diphospho-N-Acetylgalactosamine; proline betaine; 2-Aminoethanesulfinic Acid; Glycine; UDP-glucose; Anthranilic acid; Nicotinamide; ADP-ribose; N-Acetyl-5-Hydroxytryptamine; N-Acetylphenylalanine; Phosphocholine; L-Cysteine; Nicotinic Acid Adenine Dinucleotide; Glutathione Reducedform | Vitamin E; proline betaine; 9-Fluorenone; Adenine; Guanosine; Spermidine; Adipic Acid; Β-Pseudouridine; L-Threonine; Cholesterol; L-Cystine; Inosine; Dulcitol; o-Xylene; Betaine; Xanthosine; 2-Aminoethanesulfonic Acid; L-Carnosine; N-Acetyl-5-Hydroxytryptamine; L-Homoserine; Adenosine 5’-Monophosphate; Norepinephrine; DL-Benzylsuccinic acid; D-Mannitol; L-Ornithine; Shikimic Acid; D-Sorbitol | Adenosine 5’-Diphosphate; Succinic Acid; Benzoic Acid; 2-Deoxyribose 1-Phosphate; Adenine; Cytidine; Vitamin D3; D-Glucose; S-Sulfo-L-Cysteine; Adipic Acid; Glycerol 3-phosphate; Xanthosine; N-Acetylphenylalanine; 1-Methylhistidine; Phosphocholine; N-Acetylmannosamine; N-Methyl-L-Glutamate; D-Sorbitol | 2-Deoxyribose 1-Phosphate; Glucosamine; L-Asparagine Anhydrous; Glycine; Inosine; Adenine; Guanosine; D-Glucose; Vitamin E; Linoleic Acid (C18:2N6C);5-Oxoproline; Biotin; Spermidine; Hexadecanedioic acid; L-Homoserine; Adenosine 5’-Monophosphate; 2-Methylbenzoic acid; Scyllo inositol; Adenosine 5’-Diphosphate; Thiamine Monophosphate; Glycerol 3-phosphate; N-Acetylmannosamine; Cyclic Amp; Myoinositol; S-(5-Adenosy)-L-Homocysteine; Epinephrine; Furfural |
| mTOR signaling pathway | - | Adenosine 5’-Monophosphate | - | Adenosine 5’-Monophosphate |
| Neomycin, kanamycin and gentamicin biosynthesis | UDP-glucose | - | D-Glucose | D-Glucose |
| Neuroactive ligand-receptor interaction | 2-Aminoethanesulfonic Acid; L-Thyroxine; Glycine | 2-Aminoethanesulfonic Acid; Norepinephrine | Adenosine 5’-Diphosphate | Adenosine 5’-Diphosphate; Epinephrine; Glycine |
| Oocyte meiosis | - | - | - | Cyclic Amp |
| Oxidative phosphorylation | - | - | - | Adenosine 5’-Diphosphate |
| Nicotinate and nicotinamide metabolism | Nicotinic Acid Adenine Dinucleotide; Nicotinamide | - | Succinic Acid | - |
| Oxidative phosphorylation | Nicotinic Acid Adenine Dinucleotide | - | Adenosine 5’-Diphosphate; Succinic Acid | - |
| Pantothenate and CoA biosynthesis | Pantothenate; L-Cysteine | Pantothenol | Pantothenol | Pantothenol |
| Pentose phosphate pathway | - | - | D-Glucose; 2-Deoxyribose 1-Phosphate | D-Glucose; 2-Deoxyribose 1-Phosphate |
| Pentose and glucuronate interconversions | UDP-glucose | - | - | - |
| Phenylalanine metabolism | N-Acetylphenylalanine | - | Succinic Acid; Benzoic Acid; N-Acetylphenylalanine | - |
| Phenylalanine, tyrosine and tryptophan biosynthesis | Anthranilic acid | Shikimic Acid | - | - |
| Phosphonate and phosphinate metabolism | Glycine | - | - | - |
| Phosphatidylinositol signaling system | - | - | - | Myoinositol |
| Phosphonate and phosphinate metabolism | - | - | - | Glycine |
| Porphyrin and chlorophyll metabolism | Glycine; L-Threonine | L-Threonine | - | Glycine |
| Propanoate metabolism | - | - | Succinic Acid | - |
| Primary bile acid biosynthesis | Glycine; Cholesterol; 2-Aminoethanesulfonic Acid | 2-Aminoethanesulfonic Acid; Cholesterol | - | Glycine |
| Progesterone-mediated oocyte maturation | - | - | - | Cyclic Amp |
| Purine metabolism | Glycine; 2’-Deoxyadenosine-5’-Monophosphate; Inosine; ADP-ribose | Adenine;Guanosine; Adenosine 5’-Monophosphate; Xanthosine;Inosine | Adenosine 5’-Diphosphate; Xanthosine; Adenine | Guanosine; Adenine; Adenosine 5’-Monophosphate; Inosine; Glycine; Adenosine 5’-Diphosphate; Cyclic Amp |
| Pyrimidine metabolism | Uridine 5-Monophosphate; UDP-glucose | Β-Pseudouridine | Cytidine; 2-Deoxyribose 1-Phosphate | 2-Deoxyribose 1-Phosphate |
| Pyruvate metabolism | - | - | Succinic Acid | - |
| Selenocompound metabolism | L-Alanine | - | - | - |
| Starch and sucrose metabolism | UDP-glucose | - | D-Glucose | D-Glucose |
| Steroid biosynthesis | Cholesterol | Cholesterol | Vitamin D3 | - |
| Steroid hormone biosynthesis | Cholesterol | Cholesterol | Androsterone | Androsterone |
| Sulfur metabolism | L-Cysteine; 2-Aminoethanesulfonic Acid | 2-Aminoethanesulfonic Acid; L-Homoserine | Succinic Acid | L-Homoserine |
| Sulfur relay system | L-Cysteine; L-Alanine | - | - | - |
| Taurine and hypotaurine metabolism | 2-Aminoethanesulfinic Acid; L-Alanine; L-Cysteine; Guanidinoethyl Sulfonate; 2-Aminoethanesulfonic Acid | Guanidinoethyl Sulfonate; 2-Aminoethanesulfonic Acid | - | - |
| Thiamine metabolism | Glycine; L-Cysteine; Nicotinic Acid Adenine Dinucleotide | - | - | Glycine; Thiamine Monophosphate |
| Tryptophan metabolism | N-Acetyl-5-Hydroxytryptamine; Anthranilic acid | N-Acetyl-5-Hydroxytryptamine | Succinic Acid | Epinephrine |
| Tyrosine metabolism | L-Thyroxine | Norepinephrine | - | - |
| Ubiquinone and other terpenoid-quinone biosynthesis | - | Vitamin E | - | Vitamin E |
| Valine, leucine and isoleucine biosynthesis | L-Isoleucine; L-Threonine | L-Threonine | - | - |
| Valine, leucine and isoleucine degradation | L-Isoleucine | - | - | - |
| Vascular smooth muscle contraction | - | Norepinephrine | - | Cyclic Amp |

“-” represent no enriched metabolic on this pathway
