# Supplementary material for: Metabolomic Analysis of SCD during Goose Follicular Development: Implications for Lipid Metabolism
Source: Genes (Basel). 2020 Aug 26;11(9):1001. doi: 10.3390/genes11091001 (PMC7565484; doi:10.3390/genes11091001)
Supplement: Supplementary file 1 [file genes-11-01001-s001.zip › Supplementary data/Table S 4.docx]

| **S. Table 4 Metabolic pathways and significantly different metabolites are associated with lipid metabolism and enriched in the overexpressed or the knockdown groups.** | | | | | | |
| --- | --- | --- | --- | --- | --- | --- |
| Pathway | N vs. S comparison | G vs. S comparison | C vs. T comparison | C vs. F comparison | Reproductive functions reported | Reference |
| FoxO signaling pathway | - | Adenosine 5’-Monophosphate | Adenosine 5’-Diphosphate; D-Glucose | Adenosine 5’-Monophosphate; D-Glucose; Adenosine 5’-Diphosphate | FOXO1 may play a key role in granulosa cells to modulate lipid and sterol biosynthesis, thereby preventing elevated steroidogenesis during early stages of follicle development | ^1^ |
| Carbon metabolism | Glycine; L-Cysteine; L-Alanine | - | Succinic Acid | Glycine | The evidence indicating that carbon from α-linolenate and linoleate is readily recycled into newly synthesized lipids. This pathway consumes the majority of these fatty acids that is not β-oxidized as a fuel. | ^2^ |
| Glycerolipid metabolism | UDP-glucose | - | Glycerol 3-phosphate | Glycerol 3-phosphate | The regulation of lipolysis and importance of intracellular glycerolipid/fatty acid cycling within trophoblasts warrants attention. | ^3^ |
| Glycerophospholipid metabolism | Phosphocholine; Lysopc 14:0; Choline; Lysopc 18:2; Lysopc 18:1 | - | Lysopc 18:1; Phosphocholine; Lysopc 14:0; Glycerol 3-phosphate | Glycerol 3-phosphate; Diethanolamine | The findings indicate a high complexity of glycerophospholipid metabolism and a distinct acyl specificity in intact cells that are not apparent from studies *in vitro* | ^4^ |
| Starch and sucrose metabolism | UDP-glucose | - | D-Glucose | D-Glucose | Sucrose metabolism plays pivotal roles in development, stress response, and yield formation, mainly by generating a range of sugars as metabolites to fuel growth and synthesize essential compounds (including protein, cellulose, and starch) and as signals to regulate expression of microRNAs, transcription factors, and other genes and for crosstalk with hormonal, oxidative, and defense signaling | ^5^ |
| Primary bile acid biosynthesis | Glycine; Cholesterol; 2-Aminoethanesulfonic Acid | 2-Aminoethanesulfonic Acid; Cholesterol | - | Glycine | For the first time evidence that all aspects of the bile acid synthesis pathway are present in the human ovarian follicle，the functional evidence that bile acids are produced by the human follicular granulosa cells in response to cholesterol presence in the culture media. | ^6^ |
| Aminoacyl-tRNA biosynthesis | L-Threonine; Glycine; L-Alanine; L-Cysteine; L-Isoleucine | L-Threonine | - | Glycine; L-Asparagine Anhydrous | The different groups of lipids composed the high molecular complex aminoacyl-tRNA synthetases, some of them play structural role and other (prostaglandins, some phospholipids) are the regulatory components of the complex. | ^7^ |
| Biosynthesis of unsaturated fatty acids | EPA [5Z,8Z,11Z,14Z,17Z-eicosapentaenoic acid]; Linoleic Acid (C18:2N6C); Hexadecanoic Acid (C16:0) | Oleate | - | EPA [5Z,8Z,11Z,14Z,17Z-eicosapentaenoic acid]; Linoleic Acid (C18:2N6C) | Unsaturated fatty acids are found in membranes as esterified phosphor-glycerides (also known as phospholipids). | ^8^ |
| Fatty acid biosynthesis | Hexadecanoic Acid (C16:0); Palmitoleic Acid (C16:1) | Oleate | - | Palmitoleic Acid (C16:1) | Fatty acid biosynthesis and membrane lipid formation represent major metabolic energy commitments for growing bacterial cells | ^9^ |
| Ferroptosis | L-Cysteine; Glutathione Reducedform | L-Cystine; Vitamin E | - | Vitamin E | support that changing membrane properties during lipid peroxidation occur during ferroptosis and could lead to cell death | ^10^ |
| Glutathione metabolism | L-Ornithine; Spermidine; Glycine; L-Cysteine; Glutathione Reducedform | L-Ornithine; Spermidine | - | Glycine; Spermidine | When the hepatic glutathione depletion reaches a threshold level, lipid peroxidation develops and severe cellular damage is produced. | ^11^ |
| Arginine and proline metabolism | 4-Guanidinobutyric Acid; L-Ornithine; Spermidine | L-Ornithine; Spermidine | - | Spermidine | Arginine favoring lipogenesis in muscle but lipolysis in adipose tissue. proline catabolism regulates lipid metabolism. | ^12, 13^ |
| Ubiquinone and other terpenoid-quinone biosynthesis | - | Vitamin E | - | Vitamin E | Vitamin E, an antioxidant in the lipid compartment of cells (i.e., membranes) | ^14^ |
| Biotin metabolism | Biotin | - | - | Biotin | Pharmacologic doses of biotin had no effect on tissue cholesterol content or serum lipoprotein profile | ^15^ |
| mTOR signaling pathway | - | Adenosine 5’-Monophosphate | - | Adenosine 5’-Monophosphate | mTOR signaling to promote anabolic metabolism, such as protein synthesis and lipid synthesis, and to inhibit catabolic pathways, such as lysosome biogenesis and autophagy | ^16^ |
| Vascular smooth muscle contraction | - | Norepinephrine | - | Cyclic Amp | predict that therapy to lower the levels of cholesterol in the serum of patients would reduce the occurrence of cardiovascular events associated with abnormal vascular contractions | ^17^ |
| Thiamine metabolism | Glycine; L-Cysteine; Nicotinic Acid Adenine Dinucleotide | - | - | Glycine; Thiamine Monophosphate | Thiamine inhibits lipid peroxidation in rat liver microsome and free radical oxidation of oleic acidin vitro | ^18^ |
| Caffeine metabolism | - | Xanthosine | Xanthosine | - | Caffeine ingestion stimulates both lipolysis and energy expenditure | ^19^ |
| Tyrosine metabolism ^*^ | L-Thyroxine | Norepinephrine | - | - | Thyroid hormones play a role in the modulation of the FSH/LH-mediated control of granulosa cell function | ^20^ |
| Arginine biosynthesis/ D-Arginine and D-ornithine metabolism ^*^ | L-Ornithine | L-Ornithine | - | - | l-Ornithine intake caused anorexia and reductions of body weight and abdominal fat. | ^21^ |
| alpha-Linolenic acid metabolism ^*^ | Stearidonic Acid | Stearidonic Acid | - | - | The principal biological role of alpha-linolenic acid (alphaLNA; 18:3n-3) appears to be as a precursor for the synthesis of longer chain n-3 polyunsaturated fatty acids (PUFA) | ^22^ |
| Taurine and hypotaurine metabolism ^*^ | 2-Aminoethanesulfinic Acid; L-Alanine; L-Cysteine; Guanidinoethyl Sulfonate; 2-Aminoethanesulfonic Acid | Guanidinoethyl Sulfonate; 2-Aminoethanesulfonic Acid | - | - | hypotaurine might inhibit lipid peroxidation in vivo by scavenging the initiator hydroxyl radical. | ^23^ |
| Glycolysis/Gluconeogenesis ^#^ | - | - | D-Glucose | D-Glucose | In adipocytes, glucose is stored primarily as lipid | ^24^ |
| Insulin signaling pathway ^#^ | - | - | D-Glucose | Cyclic Amp; D-Glucose | The insulin signaling pathway is a highly conserved regulator of metabolism, regulating multiple physiological functions including lipid metabolism | ^25^ |
| Lysosome ^#^ | - | - | Adenosine 5’-Diphosphate | Adenosine 5’-Diphosphate | cholesterol abnormalities determine lysosomal dysfunction and endocytic traffic jam in LSDs by impairing the membrane fusion machinery. | ^26^ |

“-” represent no enriched metabolic on this pathway

“*” represent pathway is overexpress SCD [special](javascript:;) enriched

“#” represent pathway is knockdown SCD [special](javascript:;) enriched

Reference：

1. Liu, Z.; Rudd, M. D.; Hernandezgonzalez, I.; Gonzalezrobayna, I.; Fan, H.; Zeleznik, A. J.; Richards, J. S. FSH and FOXO1 regulate genes in the sterol/steroid and lipid biosynthetic pathways in granulosa cells. *Mol. Endocrinol.* **2009,** *23* (5), 649-661.

2. Cunnane, S. C.; Ryan, M. A.; Nadeau, C. R.; Bazinet, R. P.; Musaveloso, K.; Mccloy, U. J. L. Why is carbon from some polyunsaturates extensively recycled into lipid synthesis. *Lipids* **2003,** *38* (4), 477-484.

3. Pathmaperuma, A.; Mana, P.; Cheung, S. N.; Kugathas, K.; Josiah, A.; Koina, M. E.; Broomfield, A.; Delghingaroaugusto, V.; Ellwood, D.; Dahlstrom, J. E. J. P. Fatty acids alter glycerolipid metabolism and induce lipid droplet formation, syncytialisation and cytokine production in human trophoblasts with minimal glucose effect or interaction. *Placenta* **2010,** *31* (3), 230-239.

4. Holmsen, H.; Hindenes, J.; Fukami, M. H. Glycerophospholipid metabolism: Back to the future. *Thromb. Res.* **1992,** *67* (3), 313-323.

5. Ruan. Y. Sucrose Metabolism: Gateway to Diverse Carbon Use and Sugar Signaling. [*Annu. Rev. Plant. Biol.*](https://www.medsci.cn/sci/submit.do?id=331611063) 20**14,** *65* (1), 33-67.

6. Smith, L. P.; Maik, N.; Wook, Y. S.; Penzias, A. S.; Edda, T.; Anny, U. The Bile Acid Synthesis Pathway Is Present and Functional in the Human Ovary. *Plos. One.* **2009,** *4* (10), e7333-.

7. Marinello, E.; Ciccoli, L.; Leoncini, R.; Marcolongo, R.; Periccioli, E.; Vannoni, D. Relationship between lipid and purine metabolism: the behavior of fatty acids in plasma triglycerides of gouty patients. [*Adv. Exp. Med. Biol.*](https://www.medsci.cn/sci/submit.do?id=570c177) **1985,** *19* (7), 313-316.

8. Lunn, J.; Theobald, H. E. The health effects of dietary unsaturated fatty acids. [*Nutr. Bull.*](https://www.medsci.cn/sci/submit.do?id=b58f7427) **2006,** *31* (3), 178-224.

9. Menendez, J. A.; Lupu, R. Fatty acid synthase-catalyzed de novo fatty acid biosynthesis: from anabolic-energy-storage pathway in normal tissues to jack-of-all-trades in cancer cells. [*Arch. Immunol. Ther. Ex.*](https://www.medsci.cn/sci/submit.do?id=20822867) **2004,** *52* (6), 414.

10. Agmon, E.; Solon, J.; Bassereau, P.; Stockwell, B. R. Modeling the effects of lipid peroxidation during ferroptosis on membrane properties. *Sci. Rep.* **2018,** *8* (1), 5155-5155.

11. Comporti, M. Glutathione depleting agents and lipid peroxidation. [*Chem. Phys. Lipids*](https://www.medsci.cn/sci/submit.do?id=95d31440)*.* **1987**, 45, 143-169.

12. Tan, B.; Yin, Y.; Liu, Z.; Tang, W.; Xu, H.; Kong, X.; Li, X.; Yao, K.; Gu, W.; Smith, S. B. Dietary l-arginine supplementation differentially regulates expression of lipid-metabolic genes in porcine adipose tissue and skeletal muscle. *J. Nutri. Biochem*. **2011,** *22* (5), 441-445.

13. Pang, S.; Lynn, D. A.; Lo, J. Y.; Paek, J.; Curran, S. P. SKN-1 and Nrf2 couples proline catabolism with lipid metabolism during nutrient deprivation. *Nat. Commun*. **2014,** *5* (1), 5048-5048.

14. Ji, H.; Om, A. D.; Yoshimatsu, T.; Hayashi, M.; Umino, T.; Nakagawa, H.; Asano, M.; Nakagawa, A. Effect of dietary vitamins C and E fortification on lipid metabolism in red sea bream Pagrus major and black sea bream Acanthopagrus schlegeli. *Fisheries. Sci.* **2003,** *69* (5), 1001-1009.

15. Suchy, S. F.; Wolf, B. Effect of biotin deficiency and supplementation on lipid metabolism in rats: cholesterol and lipoproteins. [*Am. J. Clin. Nutr.*](https://www.medsci.cn/sci/submit.do?id=58df301) **1986,** (5), 5.

16. Kim, Y.; Guan, K. mTOR: a pharmacologic target for autophagy regulation. [*J. Clin. Invest.*](https://www.medsci.cn/sci/submit.do?id=753b3596) **2015,** *125* (1), 25-32.

17. Cox, D. A.; Cohen, M. L. Effects of oxidized low-density lipoprotein on vascular contraction and relaxation: clinical and pharmacological implications in atherosclerosis. [Pharmacol. Rev.](https://www.medsci.cn/sci/submit.do?id=cd135367) **1996,** *48* (1), 3-19.

18. Lukienko, P. I.; Melnichenko, N. G.; Zverinskii, I. V.; Zabrodskaya, S. V. Antioxidant properties of thiamine. [*B. Exp. Biol. Med.*](https://www.medsci.cn/sci/submit.do?id=20b6960) **2000,** *130* (9), 874-876.

19. Acheson, K. J.; Gremaud, G.; Meirim, I.; Montigon, F.; Krebs, Y.; Fay, L. B.; Schneiter, P.; Schindler, C.; Tappy, L. Metabolic effects of caffeine in humans: lipid oxidation or futile cycling? [*Am. J. Clin. Nutr.*](https://www.medsci.cn/sci/submit.do?id=58df301) **2004,** *79* (1), 40-46.

20. Wakim, A. N.; Polizotto, S. L.; Burholt, D. R. Augmentation by thyroxine of human granulosa cell gonadotrophin-induced steroidogenesis. [*Hum. Reprod*](https://www.medsci.cn/sci/submit.do?id=50b42765)*.* **1995,** *10* (11), 2845-2848.

21. Konishi, Y.; Koosaka, Y.; Maruyama, R.; Imanishi, K.; Kasahara, K.; Matsuda, A.; Akiduki, S.; Hishida, Y.; Kurata, Y.; Shibamoto, T. l-Ornithine intake affects sympathetic nerve outflows and reduces body weight and food intake in rats. [*Brain. Res. Bull*](https://www.medsci.cn/sci/submit.do?id=8d271201)*.* 2015, 111, 48-52.

22. Burdge, G. C.; Calder, P. C. Conversion of alpha-linolenic acid to longer-chain polyunsaturated fatty acids in human adults. [*Reprod. Nutr. Dev.*](https://www.medsci.cn/sci/submit.do?id=e37c5918) **2005,** *45* (5), 581-597.

23. Tadolini, B.; Pintus, G.; Pinna, G.; Bennardini, F.; Franconi, F. Effects of Taurine and Hypotaurine on Lipid Peroxidation. [*Biochem. Bioph. Res. Co.*](https://www.medsci.cn/sci/submit.do?id=92721030) **1995,** *213* (3), 820-826.

24. Saltiel, A. R.; Kahn, C. R. Insulin signalling and the regulation of glucose and lipid metabolism. *Nature* **2001,** *414* (6865), 799-806.

25. Xu, X.; Gopalacharyulu, P.; Seppanenlaakso, T.; Ruskeepaa, A. L.; Aye, C.; Carson, B. P.; Mora, S.; Oresic, M.; Teleman, A. A. Insulin signaling regulates fatty acid catabolism at the level of CoA activation. [*Plos. Genet.*](https://www.medsci.cn/sci/submit.do?id=ae385894) **2012,** *8* (1).

26. Fraldi, A.; Annunziata, F.; Lombardi, A.; Kaiser, H.; Medina, D. L.; Spampanato, C.; Fedele, A. O.; Polishchuk, R. S.; Sorrentino, N. C.; Simons, K. Lysosomal fusion and SNARE function are impaired by cholesterol accumulation in lysosomal storage disorders. *EMBO. J.* **2010,** *29* (21), 3607-3620.
