# Supplementary figures and images for: Metabolomic Analysis of SCD during Goose Follicular Development: Implications for Lipid Metabolism
Source: Genes (Basel). 2020 Aug 26;11(9):1001. doi: 10.3390/genes11091001 (PMC7565484; doi:10.3390/genes11091001)

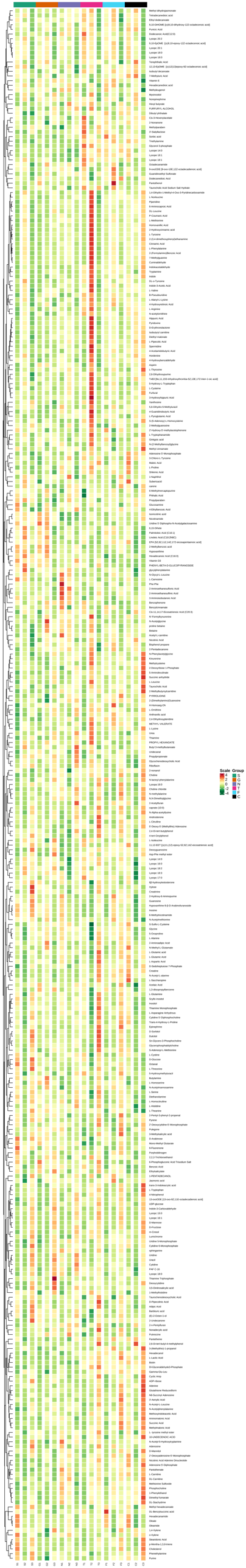

Supplement: Supplementary file 1 [file genes-11-01001-s001.zip › Supplementary data/Figure S 1.pdf]

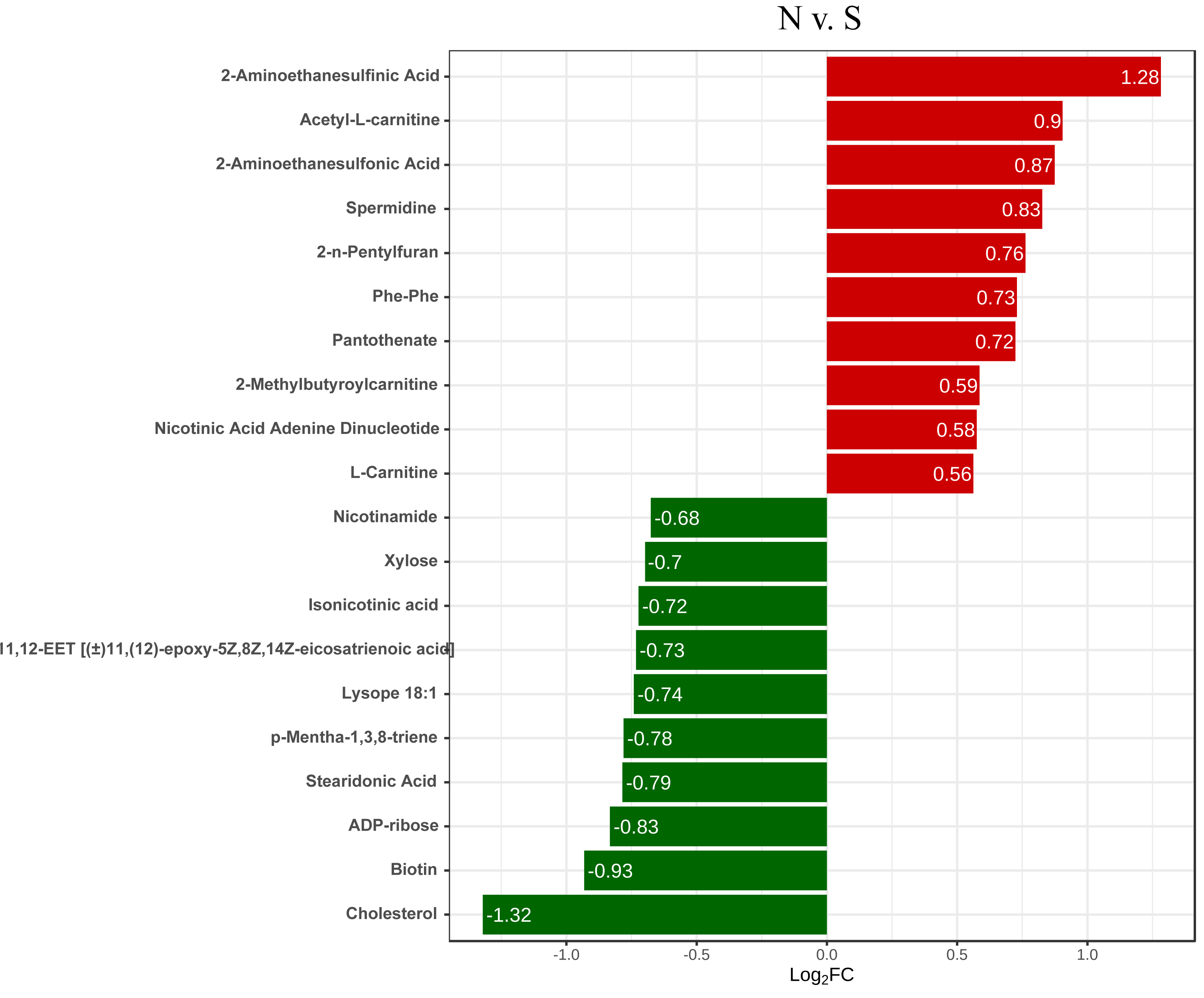

Supplement: Supplementary file 1 [file genes-11-01001-s001.zip › Supplementary data/Figure S 2A.tif]

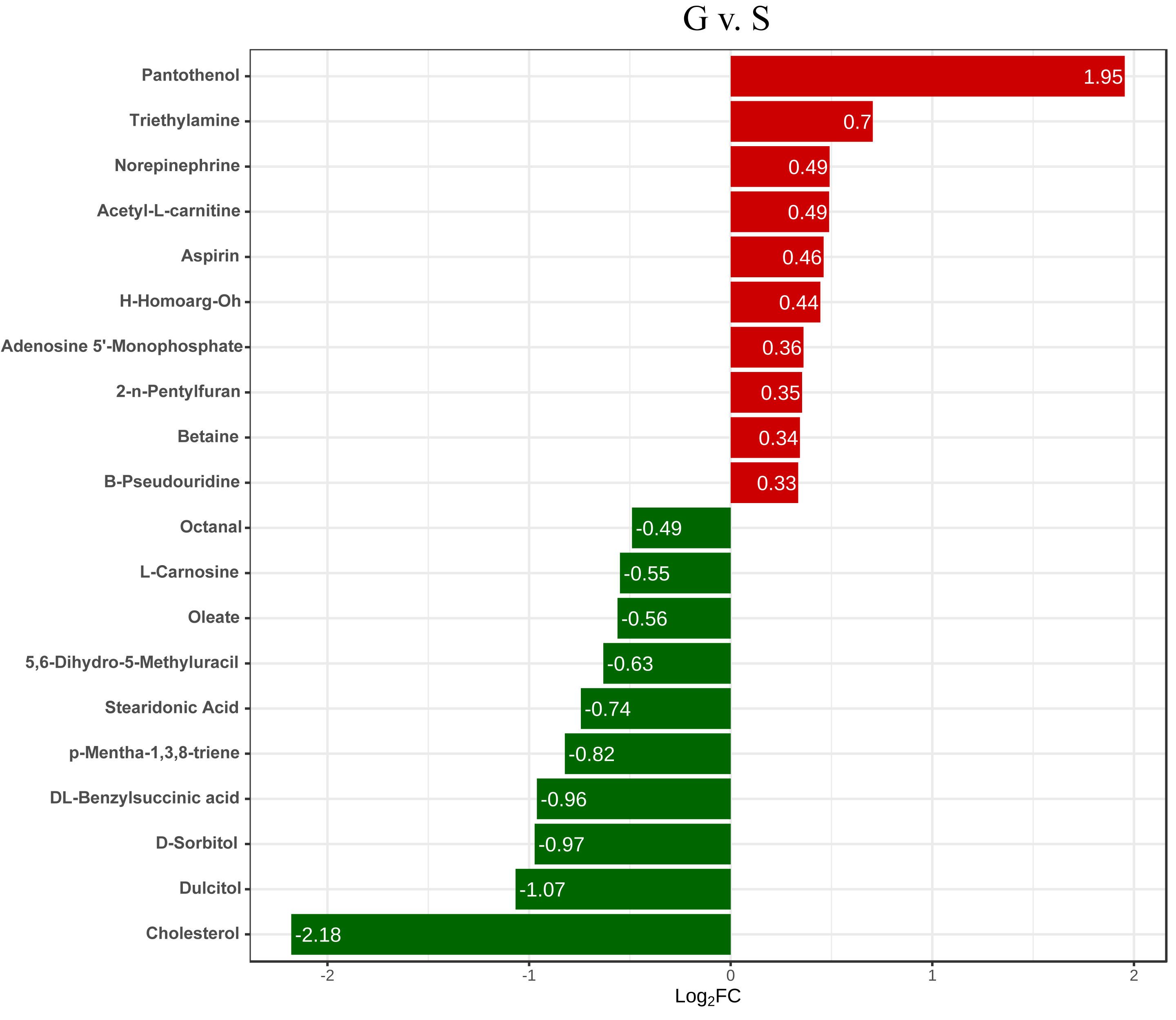

Supplement: Supplementary file 1 [file genes-11-01001-s001.zip › Supplementary data/Figure S 2B.tif]

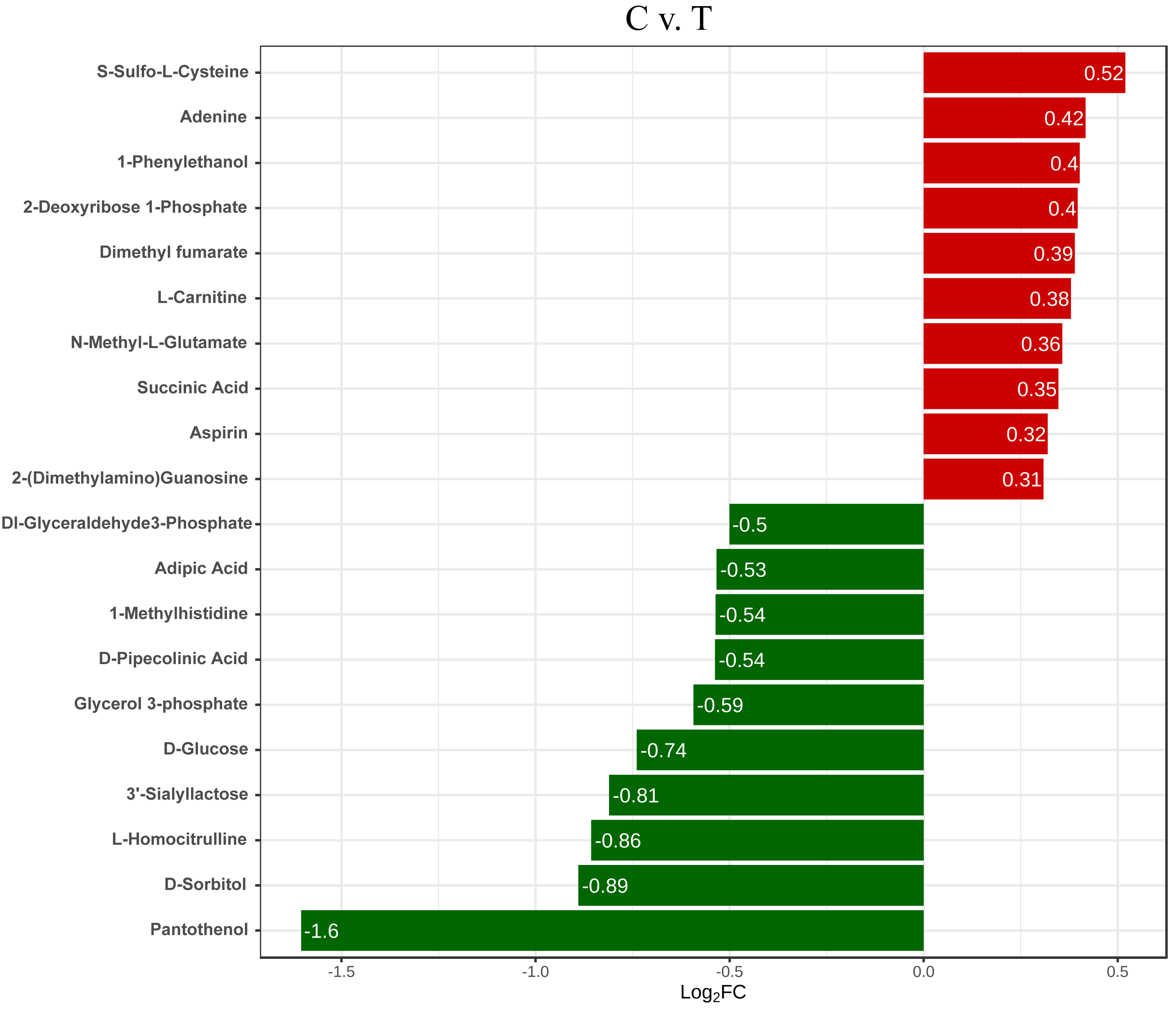

Supplement: Supplementary file 1 [file genes-11-01001-s001.zip › Supplementary data/Figure S 2C.tif]

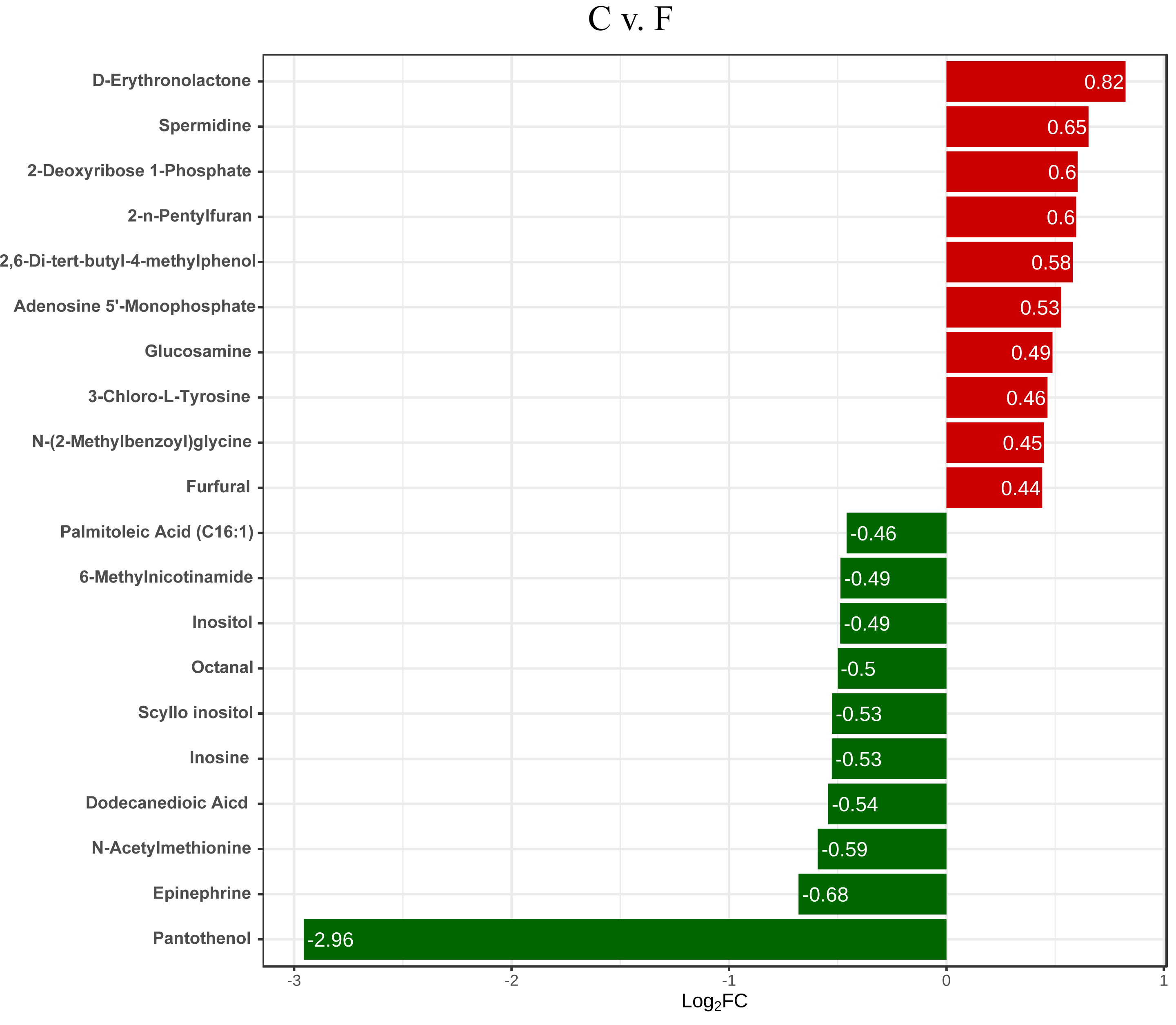

Supplement: Supplementary file 1 [file genes-11-01001-s001.zip › Supplementary data/Figure S 2D.tif]

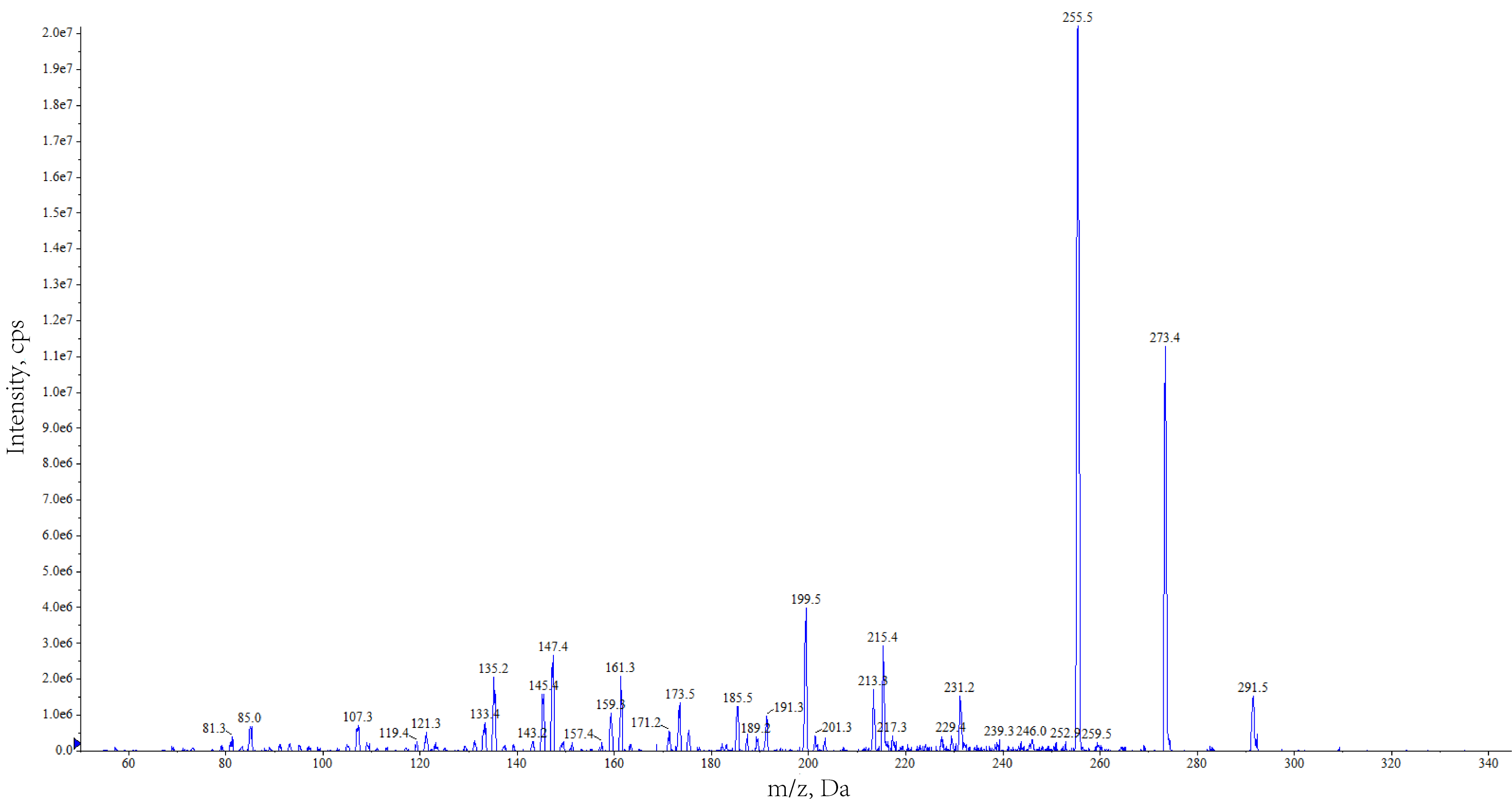

Supplement: Supplementary file 1 [file genes-11-01001-s001.zip › Supplementary data/Figure S 3A.tif]

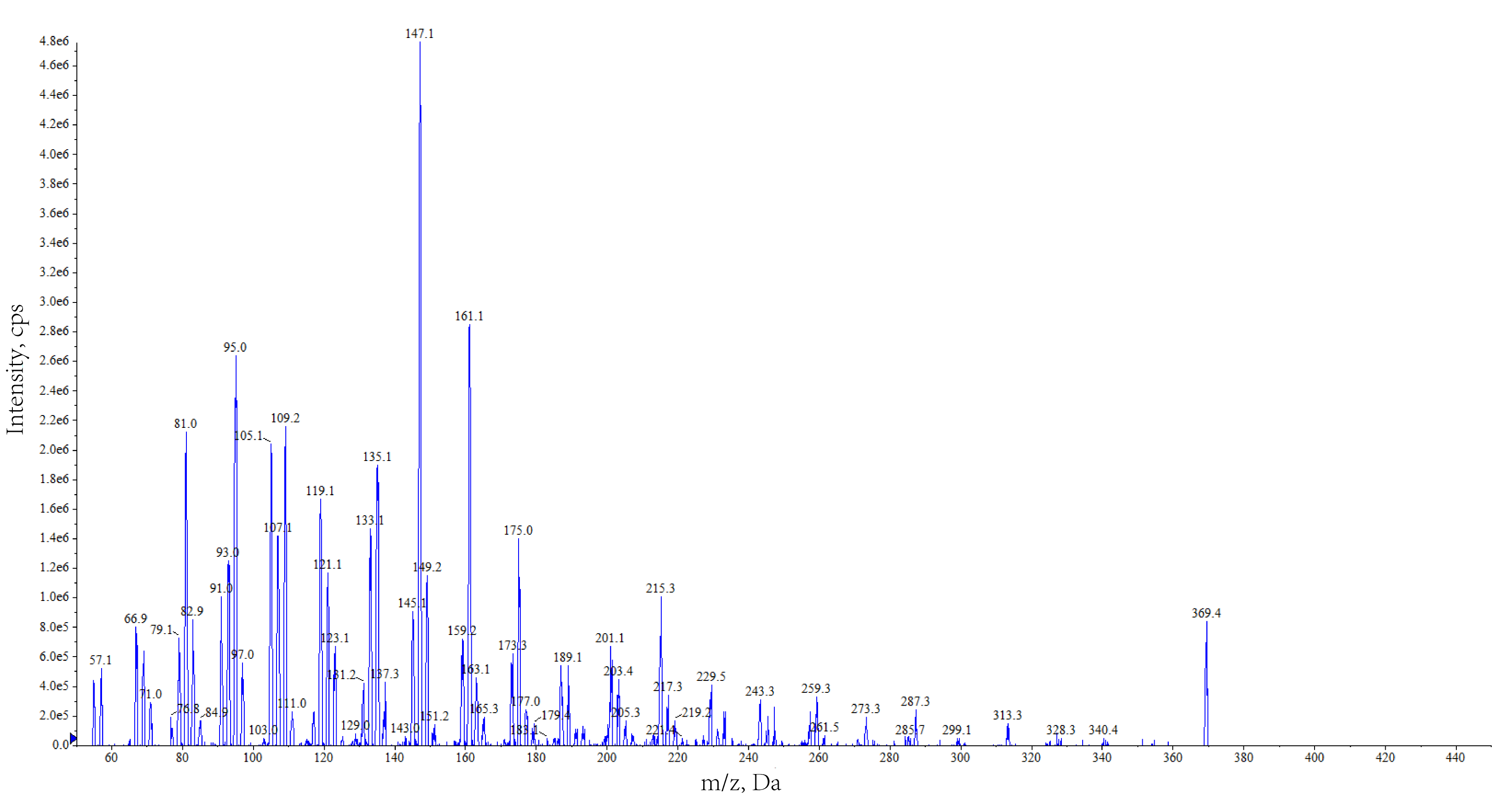

Supplement: Supplementary file 1 [file genes-11-01001-s001.zip › Supplementary data/Figure S 3B.tif]

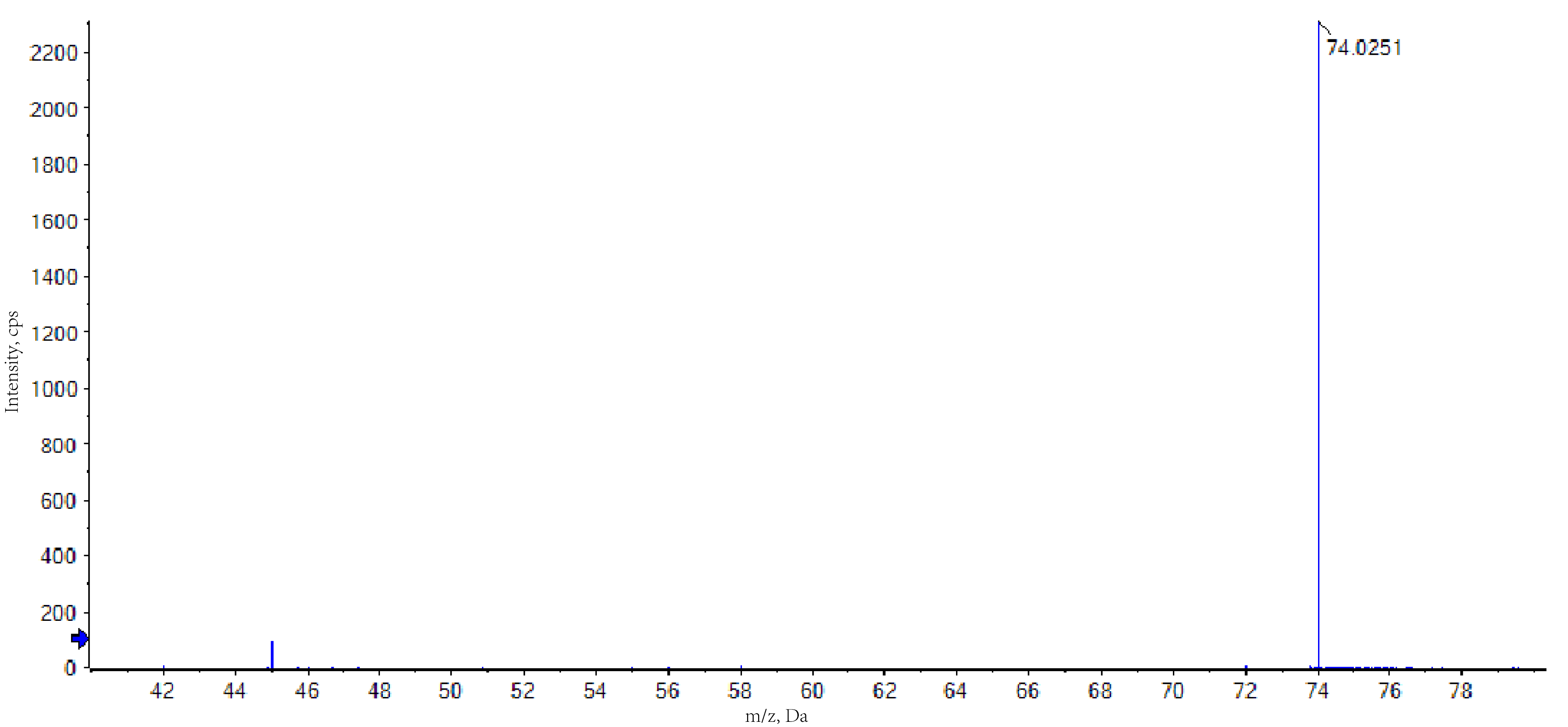

Supplement: Supplementary file 1 [file genes-11-01001-s001.zip › Supplementary data/Figure S 3C.tif]

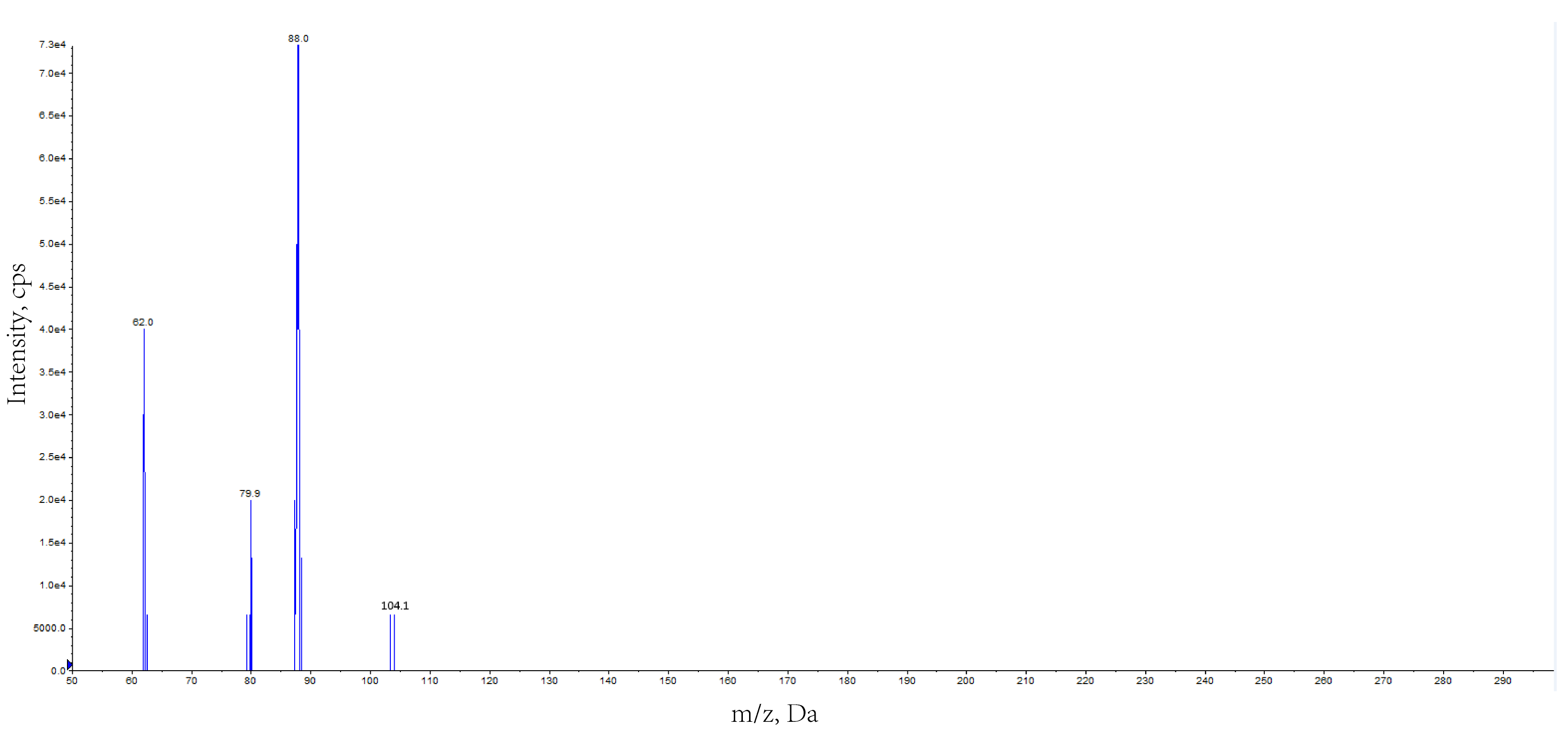

Supplement: Supplementary file 1 [file genes-11-01001-s001.zip › Supplementary data/Figure S 3D.tif]

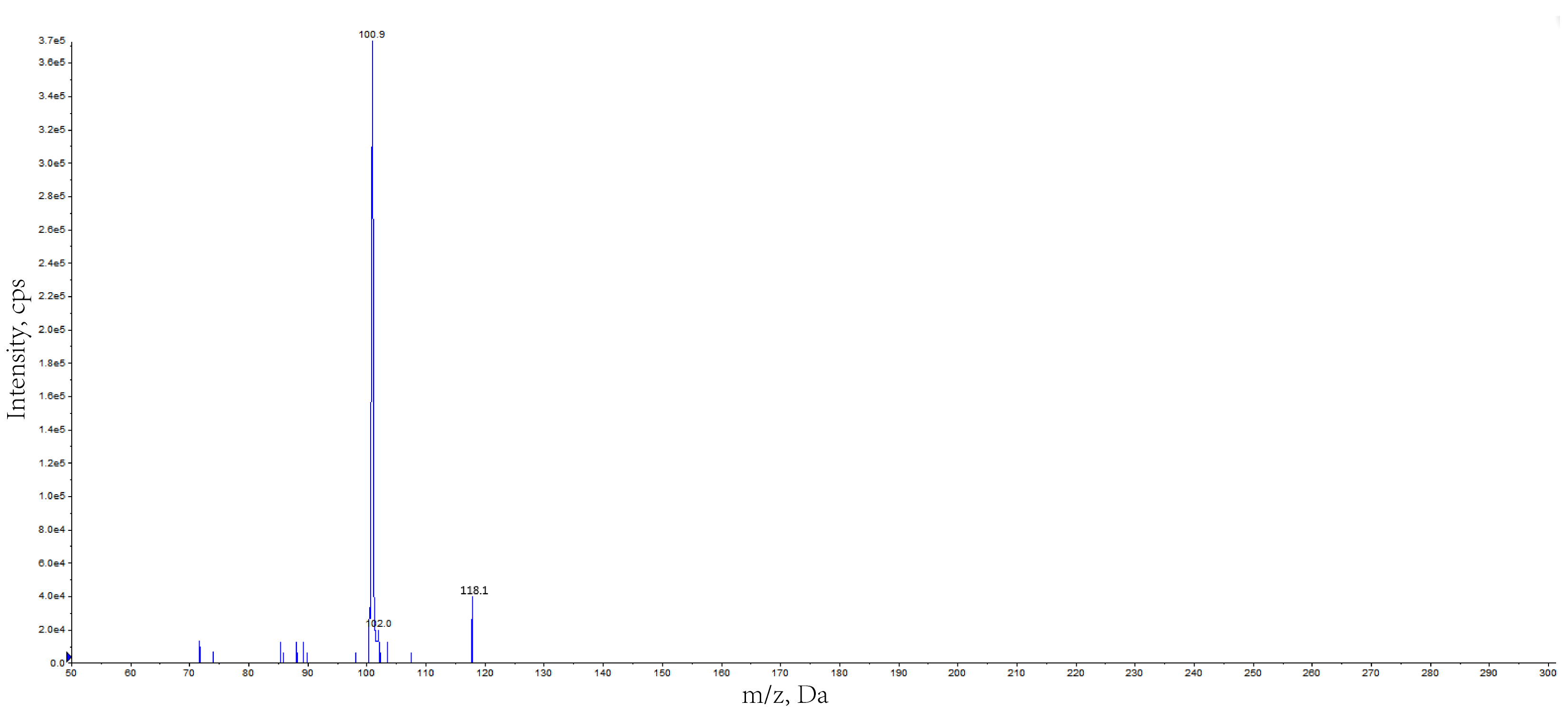

Supplement: Supplementary file 1 [file genes-11-01001-s001.zip › Supplementary data/Figure S 3E.tif]

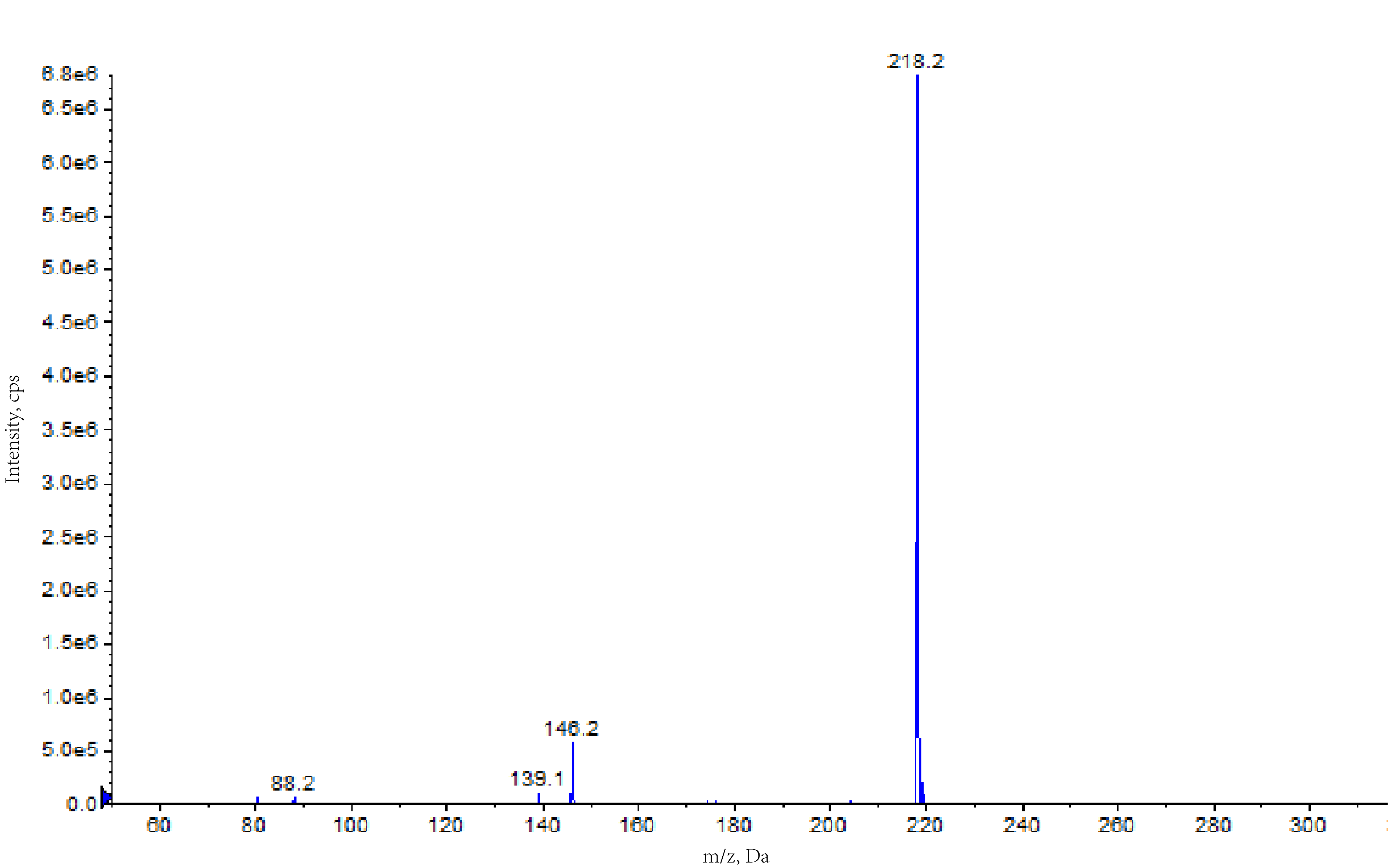

Supplement: Supplementary file 1 [file genes-11-01001-s001.zip › Supplementary data/Figure S 3F.tif]

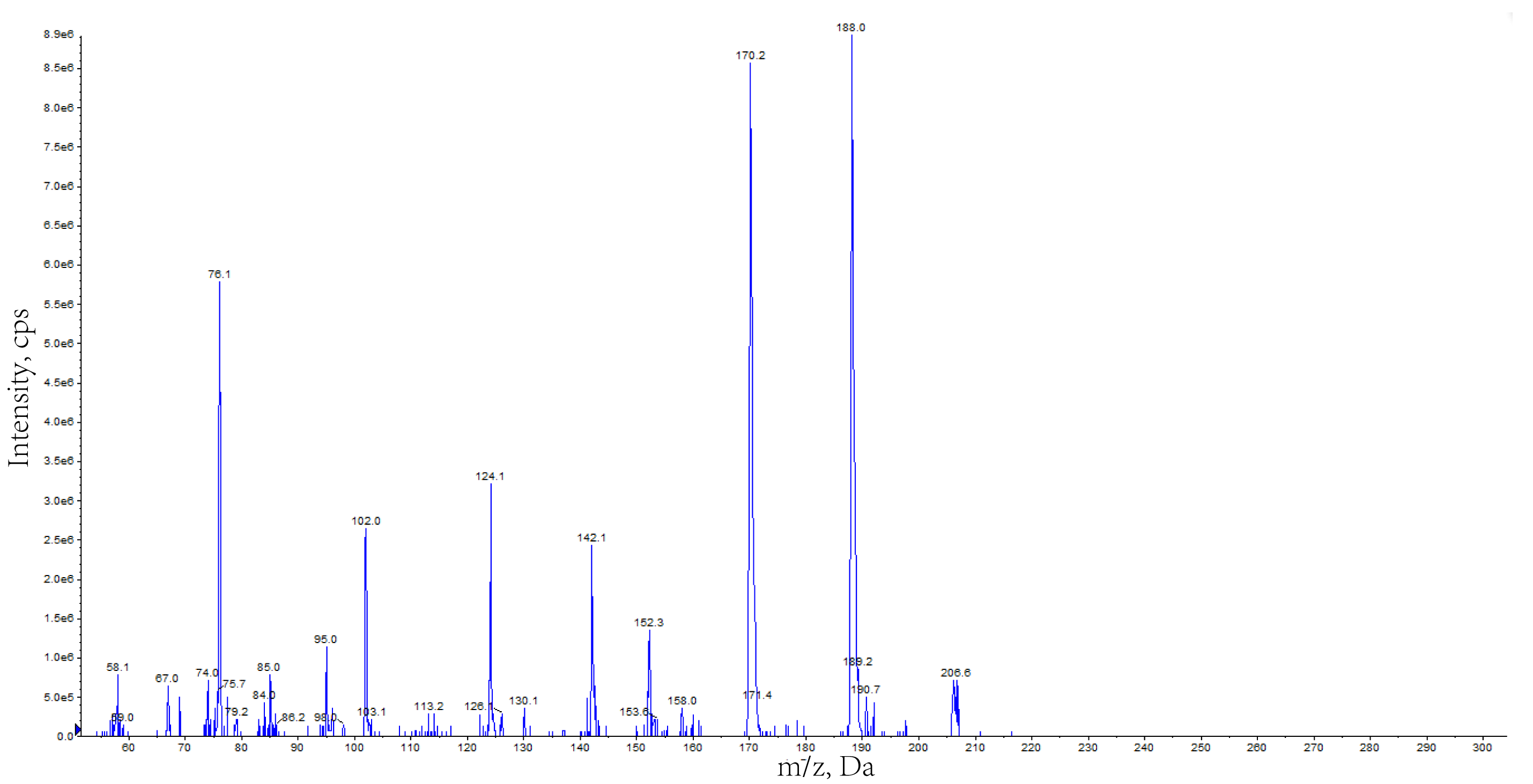

Supplement: Supplementary file 1 [file genes-11-01001-s001.zip › Supplementary data/Figure S 3G.tif]

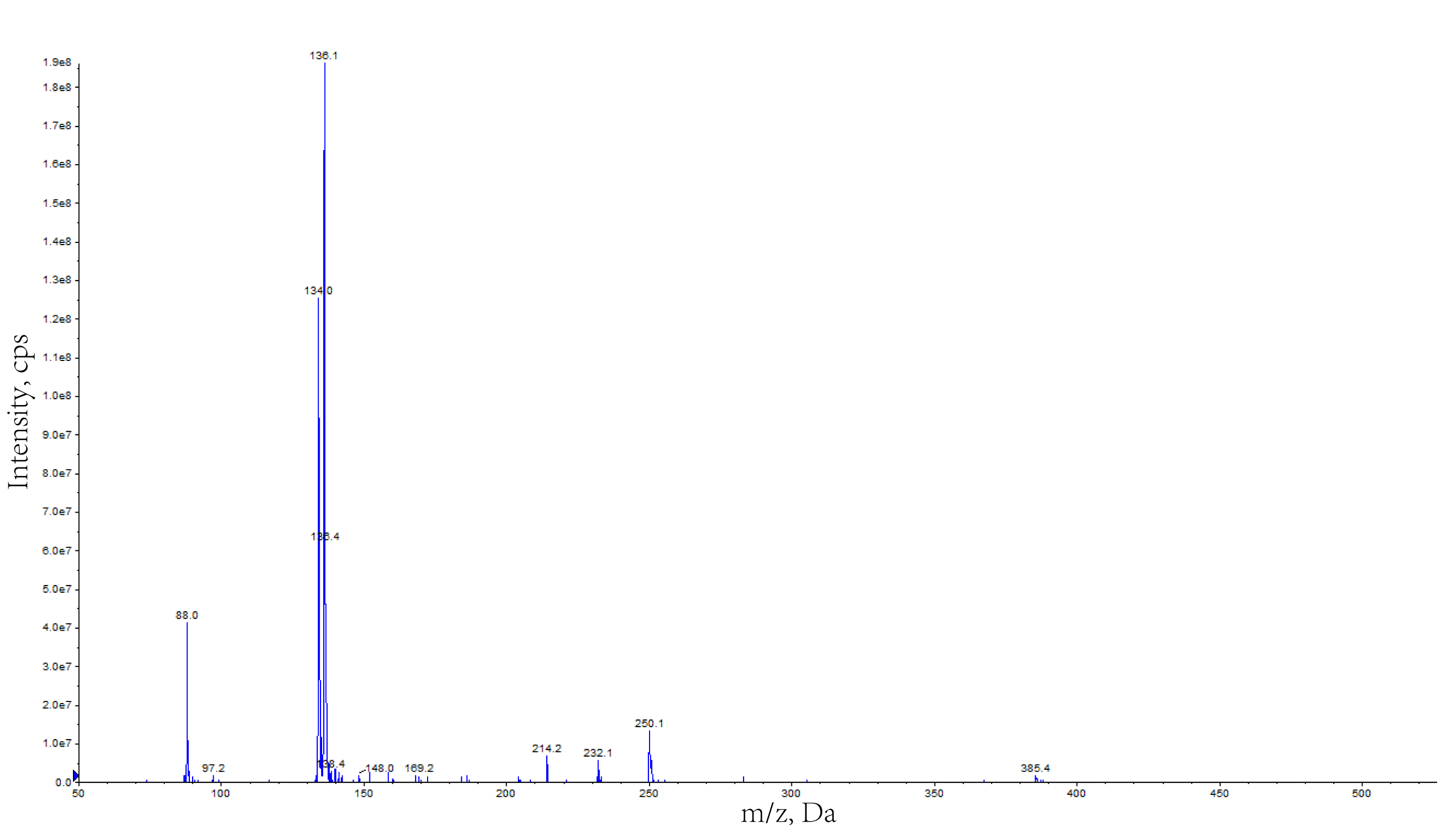

Supplement: Supplementary file 1 [file genes-11-01001-s001.zip › Supplementary data/Figure S 3H.tif]

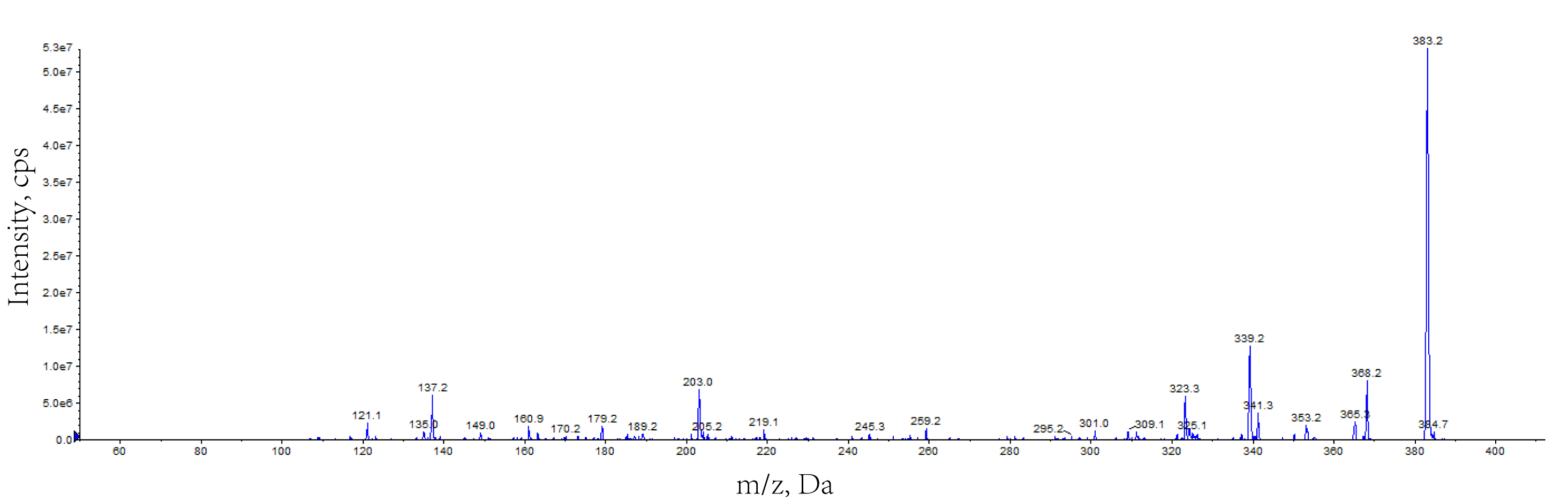

Supplement: Supplementary file 1 [file genes-11-01001-s001.zip › Supplementary data/Figure S 3I.tif]
